# The human gut microbe *Eubacterium limosum* utilizes flavodoxin over ferredoxin for lactate metabolism

**DOI:** 10.1128/aem.00643-26

**Published:** 2026-06-26

**Authors:** Saisuki Putumbaka, Nana Shao, Aaron P. Donaghy, Emma G. Harrison, Farris L. Poole, Michael P. Thorgersen, Gerrit J. Schut, Michael W. W. Adams

**Affiliations:** 1 Department of Biochemistry and Molecular Biology, University of Georgia 1355, Athens, Georgia, USA; Kyoto University, Kyoto, Japan

**Keywords:** tungsten, human gut microbiome, lactate, flavodoxin, ferredoxin

## Abstract

*Eubacterium limosum* is an abundant gut microbe that efficiently metabolizes lactate to produce the short-chain fatty acids, butyrate and acetate. The key oxidoreductase enzymes, formate dehydrogenase (FDH), carbon monoxide dehydrogenase (CODH) of the Wood-Ljungdahl pathway, pyruvate ferredoxin oxidoreductase (POR), and an electron-bifurcating tungsten-containing oxidoreductase (WOR) that detoxifies acetaldehyde produced as a byproduct of the POR reaction, are all utilized during lactate metabolism. Fermentative anaerobes typically utilize a single ferredoxin as an electron carrier for their primary pathways, which is replaced by flavodoxin under iron-limiting conditions. However, the *E. limosum* genome encodes two putative ferredoxins (Fd1 and Fd2) and three putative flavodoxins (Fld, Fld-like1, and Fld-like2). All five proteins were heterologously expressed in *Escherichia coli*, but the UV-visible absorption properties of purified Fld-like proteins 1 and 2 were not typical of canonical flavodoxins. Both POR and CODH reduced Fd1, Fd2, and Fld at comparable rates. Partially purified WOR and FDH had low activity using Fd1, Fd2, and Fld as electron carriers. With WOR, NAD-linked bifurcating activity could not be demonstrated with any of the electron carriers. Deletion mutants of *E. limosum* lacking Fd1, Fd2, or Fld exhibited similar lag phases during growth on glucose, and this increased on lactate for the Fld mutant but much less so for the Fd mutants. We conclude that *E. limosum* can utilize either Fd1, Fd2, or Fld as the primary redox protein, but that during growth on lactate, Fld plays a more prominent role than Fd1 or Fd2, even under iron-sufficient conditions.

**IMPORTANCE:**

*Eubacterium limosum* is an abundant gut microbe that is beneficial to human health due to its production of short-chain fatty acids particularly during growth on lactate. It is also of interest due to its ability to metabolize H_2_/CO_2_ and C1 substrates. It was assumed that *E. limosum*, like other fermentative anaerobes, utilizes a single ferredoxin as an electron carrier during primary carbon metabolism and only utilizes flavodoxin under iron-limited conditions. However, we show here that this organism utilizes flavodoxin as its main electron carrier. This may be a significant advantage in the gut environment where competition with the host and with other gut microbes for iron is intense. This may have both biotechnological and health implications for this organism.

## INTRODUCTION

The human gastrointestinal microbiome is a complex ecosystem that significantly impacts host health (1). Host diet, age, lifestyle, and genetics can have an impact on the gut microbiome composition, and in turn, microbiome composition is correlated with diseases and health conditions, for example, Crohn's disease, Alzheimer's, and colorectal cancer (2–6). Metabolites, such as short-chain fatty acids (SCFAs), produced by organisms in the gut microbiome are taken up by the epithelium and act as signaling molecules that help improve and maintain intestinal barrier function, glucose homeostasis, lipid metabolism, and the immune system (7). *Eubacterium limosum* is an abundant member of the gut microbiome that is involved in suppressing inflammation, improving immune system function, and increasing intestinal mucosal integrity (8, 9). *E. limosum* has a dynamic metabolism and grows on a variety of compounds, including glucose, H_2_/CO_2_, carnitine, and lactate. The catabolism of lactate, derived from the host's diet, is especially beneficial in the gut environment as lactate buildup leads to acidosis, neurotoxicity, and cardiac arrhythmia (10). Moreover, *E. limosum* converts lactate to the SCFAs acetate and butyrate, both of which have been found to promote a healthy immune system and reduce inflammation (9, 11).

In a recent exploration of lactate metabolism in *E. limosum*, we found that lactate is catabolized into equal parts butyrate and acetate through the involvement of a complex metabolic pathway that contains many oxidoreductase-type enzymes, two of which unexpectedly utilize the metal tungsten (W) rather than molybdenum (Mo) (12). The oxidoreductases include pyruvate ferredoxin oxidoreductase (POR), a W-containing formate dehydrogenase (FDH), and carbon monoxide dehydrogenase/acetyl CoA synthetase (CODH), together with three electron-bifurcating enzymes, a W-containing aldehyde oxidoreductase (WOR), butyryl-CoA dehydrogenase (BCD), and lactate dehydrogenase (LCT). FDH catalyzes the first step of the Wood-Ljungdahl pathway and converts CO_2_ to formate (13, 14), while CODH reduces CO_2_ to the carbonyl group of acetyl-CoA (15). Both enzymes are assumed to use the iron sulfur-containing redox protein ferredoxin as the electron donor. POR catalyzes the oxidation of pyruvate and coenzyme A to form acetyl-CoA coupled to the reduction of ferredoxin (16). The three bifurcating enzymes all simultaneously use both ferredoxin and NAD as electron carriers. LCT oxidizes lactate to pyruvate (17, 18), BCD reduces crotonyl-CoA to butyryl-CoA (19), and WOR is proposed to oxidize the acetaldehyde that is produced as a side-product of the POR reaction (20). The structure of a close homolog of *E. limosum* WOR was recently determined and was found to have a unique quaternary structure including nanowire-type features (21). *E. limosum* contains a second W-containing aldehyde-oxidizing oxidoreductase (WOR2) that is non-bifurcating and presumably uses ferredoxin as the sole electron carrier, but WOR2 is not involved in lactate metabolism (12).

In *E. limosum,* all of the oxidoreductases involved in lactate catabolism, namely, POR, FDH, BCD, WOR, CODH, and LCT, are assumed to involve ferredoxin (Fd) as the electron carrier, with the bifurcating enzymes also utilizing NAD (12). However, none of these *E. limosum* enzymes have been characterized, and this assumption is based on what is known about homologous enzymes in other fermentative anaerobes that have been studied, which typically contain a single Fd that serves such a role (22–24). Such oxidoreductases are routinely assayed *in vitro* using artificial dyes as the electron carrier, and when Fd is used in such activity assays, it is rarely from the same source as the enzyme, often being replaced with the very well characterized *Clostridium pasteurianum* Fd (18, 25, 26). Fds are acidic monomeric proteins of ~8 kDa that contain iron-sulfur clusters and are classified according to the type and number of clusters they contain, either [2Fe-2S], [3Fe-4S], and/or [4Fe-4S] forms (27). Fds containing one [4Fe-4S] (designated 4Fe-Fds, e.g., in *Pyrococcus furiosus* [28]) or two [4Fe-4S] clusters (8Fe-Fds, e.g., *C. pasteurianum* [29]) are typically involved in anaerobic metabolism, although there are examples where [2Fe-2S] cluster-containing Fds (2Fe-Fds), for example, *Halobacterium salinarum* (30), or Fds containing more than two 4Fe-clusters, so-called polyferredoxins, for example, *Methanobacterium thermoautotrophicum* (31), are utilized. Iron sulfur clusters catalyze the transfer of one electron; hence, 4Fe-Fds and 8Fe-Fds are one- and two-electron carriers, respectively. It is not known why some organisms use 4Fe-Fds and others 8Fe-Fds.

Fds containing [4Fe-4S] clusters are thought to be the earliest electron carrier proteins to have evolved, but the rise in global oxygen levels led to a decrease in bioavailable iron (Fe) and most likely to the evolution of the flavin-containing redox proteins known as flavodoxins (Flds) (32). Flds are also acidic monomeric proteins that are slightly larger than the typical Fd (15–22 kDa) and contain a single flavin mononucleotide (FMN). They are usually used as the dominant electron carrier in anaerobes under Fe-limiting conditions, such as with *Peptostreptococcus elsdenii* (33) and *Acidaminococcus fermentans* (34). Canonical Flds have three redox states that involve a step-wise two-electron reaction of the FMN in which the oxidized (OX) state is reduced by one electron to a partially reduced, semiquinone (SQ) state and then, after a second one-electron transfer, to the fully reduced, hydroquinone (HQ) state (35, 36). The SQ state of Fld is characteristically stable under physiological conditions, and Flds typically cycle between the SQ and HQ states, acting as one-electron carriers similar to Fd (37).

Surprisingly, we found that the genome of *E. limosum* encodes five putative electron transfer proteins, two ferredoxins, and three flavodoxins (Table 1). Moreover, all five genes are expressed based on RNAseq data when *E. limosum* is grown on glucose or lactate, even though the cells are not limited for iron (12). Accordingly, when a cytoplasmic extract of *E. limosum* was fractionated by anion exchange chromatography, there was no large Fe-containing peak eluting at a high salt concentration characteristic of a single major ferredoxin, as is typically observed with anaerobes, such as with *Acetomicrobium mobile* (12, 38). Herein, our goal was to further increase our understanding of human health-related lactate metabolism by *E. limosum* by determining which of these five redox proteins are involved in SCFA production. We used a combination of comparative genomics, transcriptomics, UV-visible absorption spectroscopy, enzymatic assays of the key oxidoreductases POR, CODH, FDH, and WOR, and phenotypic analysis of deletion mutants. We conclude that Fld, as well as Fd, are utilized as electron carriers by *E. limosum*, which may provide an advantage for survival in the competitive gut environment.

## RESULTS

### Native redox proteins in *E. limosum*

**Fig 1:**
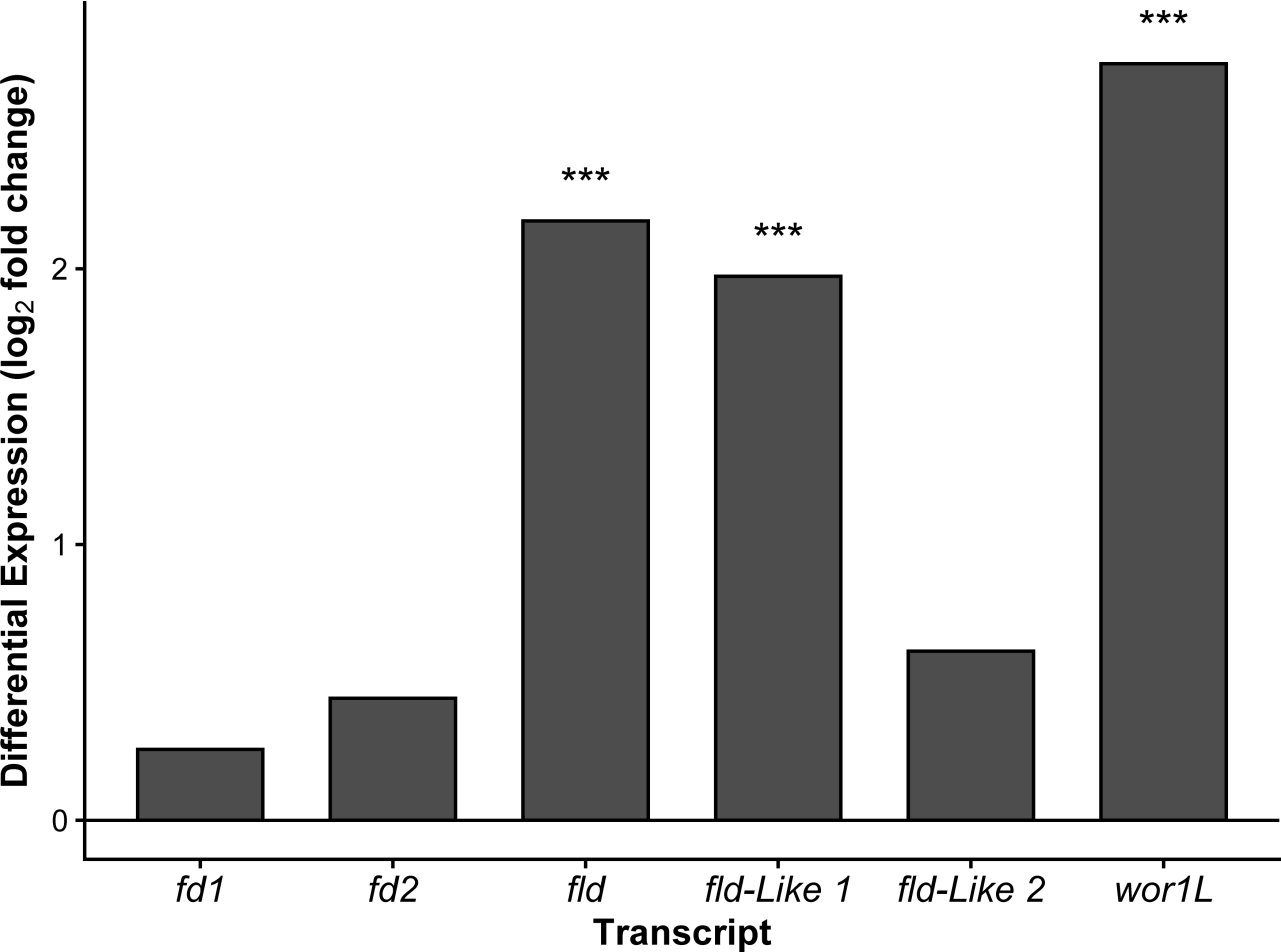
Differential expression of key genes from *E. limosum* cells grown on lactate versus glucose. RNA-Seq data were obtained from biological triplicate cultures that were grown with either lactate or glucose as the carbon source (12). Positive values indicate increased expression when grown on lactate. Significant expression changes (***) are indicated based on *P* values < 0.001.

Genome analysis of *E. limosum* revealed genes encoding two putative ferredoxins and three putative flavodoxins (Table 1). The sizes and pI values are consistent with these assignments, and the genes are not part of operons as they do not overlap with other genes, all of which are more than 16 bp distant (Fig. S1). Based on sequence analysis and the consensus spacing of cysteine residues that coordinate [4Fe-4S] clusters, one Fd, designated Fd1 (B2M23_RS10700), contains a single [4Fe-4S] cluster, while the other, designated Fd2 (B2M23_RS02975), has two [4Fe-4S] clusters. InterPro analysis confirmed the [4Fe-4S] cluster-binding domains (IPR017896) in both Fds. One of the Flds was a canonical Fld (B2M23_RS20265) while the other two were designated as Fld-like, namely, Fld-like1 (B2M23_RS01705) and Fld-like2 (B2M23_RS10125), since, based on InterPro analysis, only Fld had an assigned molecular function of electron transfer (GO:0009055). However, InterPro found that all three of these Flds were predicted to contain FMN-binding domains that were labeled as flavodoxin-like (GO:0010181; IPR008254). Previous RNA sequencing data showed that the genes encoding all five of the putative redox carrier proteins in the *E. limosum* genome are expressed during growth on both glucose and lactate, with those encoding the Fld and Fld-like1 being significantly more highly expressed on lactate (12) (Fig. 1), suggesting a role for these proteins in lactate metabolism.

**TABLE 1:** Putative ferredoxins and flavodoxins in *E. limosum^a^*

Protein	Locus	MW (kDa)	Redox cofactor	pI	Purification tag
Ferredoxin 1 (Fd1)	B2M23_RS10700	6.6	1× [4Fe-4S]	3.37	No tag
Ferredoxin 2 (Fd2)	B2M23_RS02975	5.8	2× [4Fe-4S]	3.38	C-terminal Strep II
Flavodoxin (Fld)	B2M23_RS20265	15.8	FMN	4.13	C-terminal Strep II
Flavodoxin-like 1 (Fld-like1)	B2M23_RS01705	17.2	FMN	5.21	C-terminal Strep II
Flavodoxin-like 2 (Fld-like2)	B2M23_RS10125	16.7	Unknown	4.69	C-terminal Strep II

^*a*^ For each putative electron carrier, the locus tag within the *E. limosum* genome, molecular weight (MW) in kDa, the redox cofactor, the isoelectric point (pI), and how the protein was purified (with or without a Strep II tag) are listed.

### Pangenome analysis of ferredoxins and flavodoxins in *Eubacterium*

To give a perspective on how unique *E. limosum* might be in containing five putative redox proteins, a phylogenetic tree was constructed of 350 clostridia-like strains (Table S1). These encompassed the diverse *Eubacterium* genus from the orders Eubacteriales, Peptostreptococcales, and Lachnospirales using 49 universal genes defined by Clusters of Orthologous Groups (COGs) gene family (39). Genomes of the *Eubacterium* genus that fit the quality control metrics were selected first, followed by those of other genera that were phylogenetically located between or adjacent to the various disparate *Eubacterium* groupings. The tree had three main branches (labeled 1–3); however, genus-level annotations did not fully account for species localization on the phylogenetic tree (Fig. S2). For example, *Eubacterium* species can be found in multiple different groupings in all three tree branches and, likewise, species of the *Ruminococcus* genus are found in groupings within both Branches 2 and 3. The *Eubacterium* genus is phylogenetically and metabolically diverse, making the taxonomic grouping of this genus difficult, which is why so many groupings have *Eubacterium* species with different features (8). To simplify the analysis, we subdivided the phylogenetic tree by selecting 24 specific groups (labeled a through x) that each include closely related microorganisms. *E. limosum* is located in Group g of Branch 1, which is composed of strains of the species *limosum*, *callanderi*, and *maltosivorans* of the *Eubacterium* genus (Table S1).

**Fig 2:**
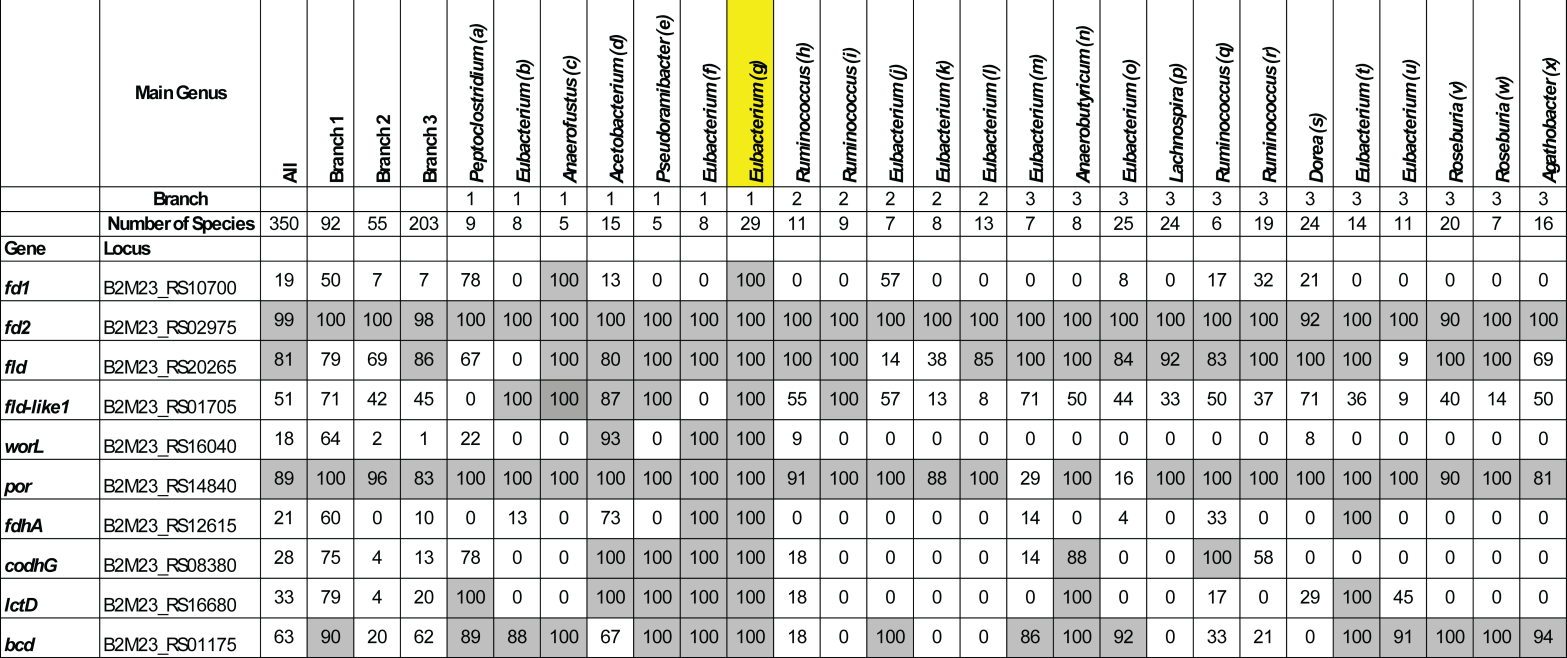
Distribution of genes encoding ferredoxin, flavodoxin, and relevant oxidoreductases in clostridia-like strains. Column groupings of organisms were selected using a phylogenetic tree of 350 clostridia-like strains (Table S1) that was constructed using multiple sequence alignments of COGs for 49 core universal genes. The incidence of genes within species of the phylogenetic tree was determined using a pangenome of the 350 strains (see Materials and Methods). The numbers within the table are the percentage of organisms in either all the organisms, each branch, or each grouping (a–x), with those higher ≥80% shaded gray. Due to the diversity of the *Eubacterium* strains, different species and strains are present in more than one group. *E. limosum* is found in Group g (highlighted in yellow).

A pangenome was constructed to determine the distribution of genes encoding Fd, Fld, and relevant oxidoreductases in microorganisms related to *E. limosum* that were included in the phylogenetic tree. All the closest related organisms to *E. limosum* in Group g contain genes encoding the same five putative redox proteins, Fd1, Fd2, Fld, Fld-like1, and Fld-like2 (Fig. 2). Looking beyond Group g to the rest of these 350 related microorganisms, the 8Fe-Fd2 appears to be the main Fd as its gene is much more widespread (in 99% of the strains) than the 4Fe-Fd1 gene (19%: Fig. 2). Fld encoding genes are also very prevalent in all of these microorganisms, with homologs in 81% of the strains Fig. 2. Of note, the sequences of *fld* and *fld-like2* are highly similar and cannot be distinguished from each other in this pangenome analysis. However, Fld-like1 has an insertion of 20 amino acids near the FMN binding site not seen in Fld and Fld-like2 and is a so-called “long” Fld (40, 41). Long Fld-like1 proteins can be distinguished from Fld (and Fld-like2), and these are encoded in 51% of the 350 strains. Hence, about half of the 350 strains encode both a conventional Fld and a long Fld (Fld-like1). All members of Group g, which includes *E. limosum,* contain two Fld genes corresponding to *fld* and *fld-like2* in addition to the *fld-like1* gene. Outside of Group g, only a few *Acetomicrobium* strains (Group d) and one *Anaerobutyricum* strain (Group n) have genes encoding two Fld proteins of the *fld/fld-like2* pangenome feature (Fig. S3). There are 16 other distinct pangenome *fld-like* features in the pangenome that are far less widespread than *fld/fld-like2* (found in 82% of all genomes) and *fld-like1* (found in 51% of all genomes), the most abundant of which is in 26% of the genomes. These 16 other features are distinguished from the *fld/fld-like2* and *fld-like1* features and each other by having <80% amino acid identity and/or <80% coverage, splitting them into separate pangenome features. Two of these 16 features have representatives in Group g that are annotated as hypothetical flavodoxin family proteins, and the rest have no Group g representatives and are not discussed further in the manuscript. In contrast, other *fd* features beyond *fd1* and *fd2* are not present in the pangenome.

*E. limosum* has multiple genes that encode oxidoreductase enzymes that catalyze electron transfer reactions to or from a metabolite that nominally require Fd as the electron carrier. We previously showed that lactate metabolism in *E. limosum* utilized W rather than Mo and involves two tungstoenzymes, FDH and the electron-bifurcating WOR (12). Other lactate metabolism related putative Fd-dependent enzymes include POR, CODH, LCT, and BCD. Interestingly, the genes encoding the catalytic subunits of all six of these enzymes (*fdhA*, *worL*, *por*, *codhG*, *lctD*, and *bcd*) are present in all of *E. limosum*-related microorganisms of Group g (Fig. 2). Some of these enzymes, like POR, are part of central carbon metabolism and are present in most of the 350 species within the pangenome (89% of total strains), while others have more specialized roles. For example, only 11% of the strains have a bifurcating WOR, while 7% have a non-bifurcating WOR (Fig. S4), and 33% have a bifurcating LCT. In any event, all 29 species in Group g contain all six oxidoreductase enzymes (FDH, WOR, POR, CODH, LCT, and BCD) and all five redox proteins (Fd1, Fd2, Fld, Fld-like1, and Fld-like2).

### Characterization of recombinant ferredoxins and flavodoxins from *E. limosum*

**Fig 3:**
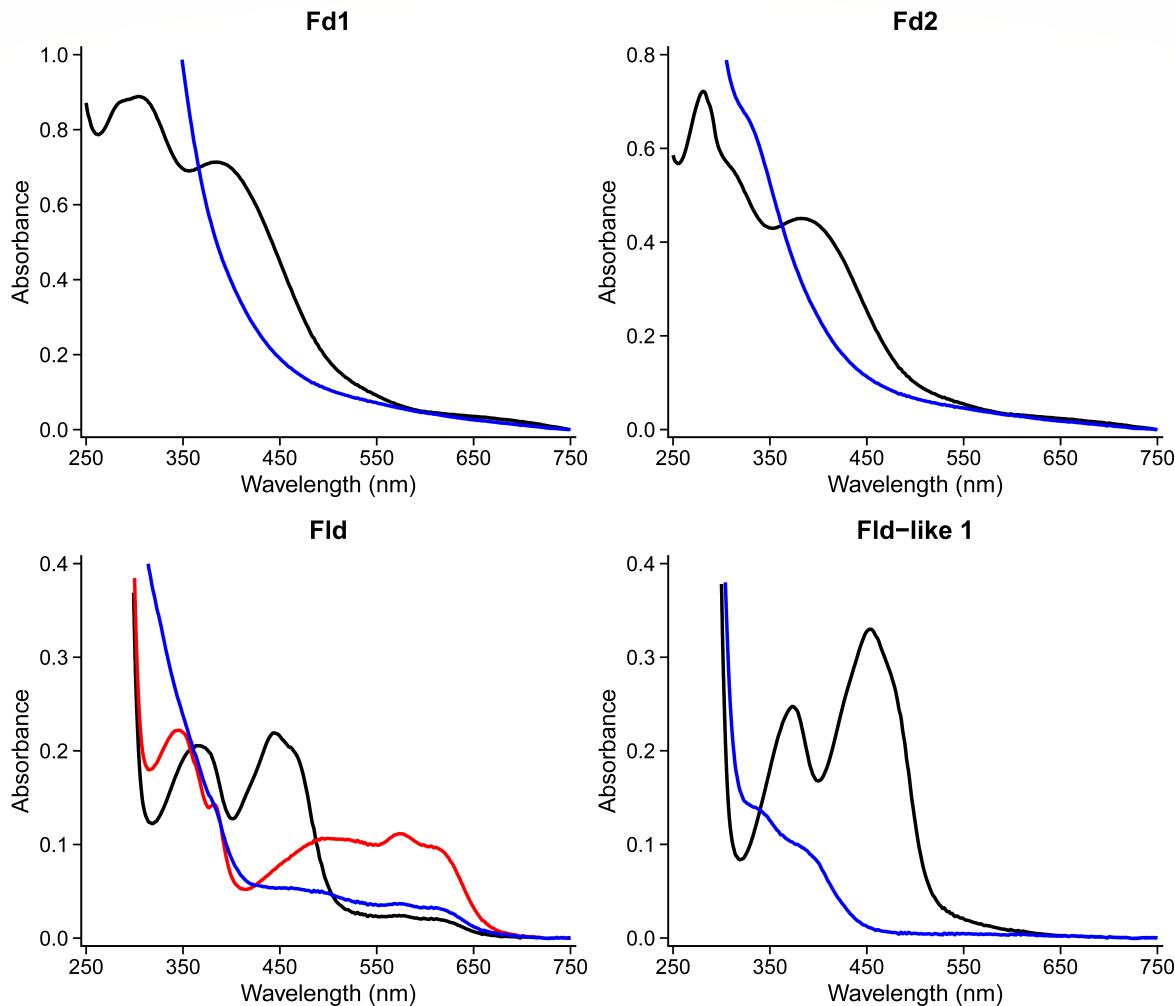
UV-visible spectra of heterologously expressed Fd1, Fd2, Fld, and Fld-like1. Spectra of proteins in the oxidized state (black line) are shown along with those of the fully reduced states after titration with Ti (III) citrate (blue line). Titration spectra are shown in Fig. S5. In the case of Fld, the spectrum of the stable SQ state is also depicted (red line). The absorption maxima of the oxidized state for Fd1 (385 nm) and Fd2 (384 nm) are characteristic of ferredoxins containing [4Fe-4S] clusters. The Fld exhibited characteristic absorption maxima in its oxidized state (364 and 444 nm) and SQ state (573 nm). After reconstitution with FMN, Fld-like1 had absorption maxima at 380 and 460 nm, but the SQ state was not observed upon reduction.

In order to biochemically and spectroscopically characterize each of the putative Fds and Flds from *E. limosum,* we heterologously expressed the genes encoding all five redox proteins in *Escherichia coli* and purified the recombinant forms. Fd1 was purified without using a tag based on its size and acidic nature, while Fd2 was purified using a Strep-tag (Table 1). Both Fds exhibited the expected broad visible absorption from their [4Fe-4S] clusters, with absorption maxima at 385 nm for Fd1 and 384 nm for Fd2, and neither protein had to be reconstituted (42, 43). The [4Fe-4S] clusters in both Fds could be chemically reduced by Ti (III) citrate, with the characteristic decrease observed in the visible absorbance between 350 and 500 nm (Fig. 3; Fig. S5).

Fld, Fld-like1, and Fld-like2 were also purified using a Strep-tag (Table 1). Purified Fld was yellow in color, and its UV-visible spectra had peaks at 364 and 444 nm, characteristic of oxidized and properly incorporated FMN, and it did not require reconstitution. Stepwise reduction with Ti (III) citrate revealed a stable intermediate SQ state with a broad peak between 450 and 650 nm (maximum at 573 nm), and then full reduction to the HQ state (Fig. 3; Fig. S5), all properties typical of a conventional Fld (35, 36). In contrast, purified Fld-like1 was colorless, lacking FMN as purified, but could be reconstituted with FMN. The UV-visible spectra of the oxidized protein exhibited peaks at 380 and 460 nm, but when it was titrated with Ti (III) citrate, an absorption peak near 620 nm corresponding to the SQ state was not observed (Fig. 3; Fig. S5). These data show that Fld-like1 is not a canonical flavodoxin, all of which have a stable semiquinone state. Similarly, purified Fld-like2 was colorless, and attempts to reconstitute it with either FMN or FAD were unsuccessful based on the visible absorption of the resulting protein (it remained colorless after removing excess FMN and FAD). Therefore, Fld-like1 and Fld-like2 were not characterized further in this study.

### Purification of ferredoxin and flavodoxin natively produced by *E. limosum*

**Fig 4:**
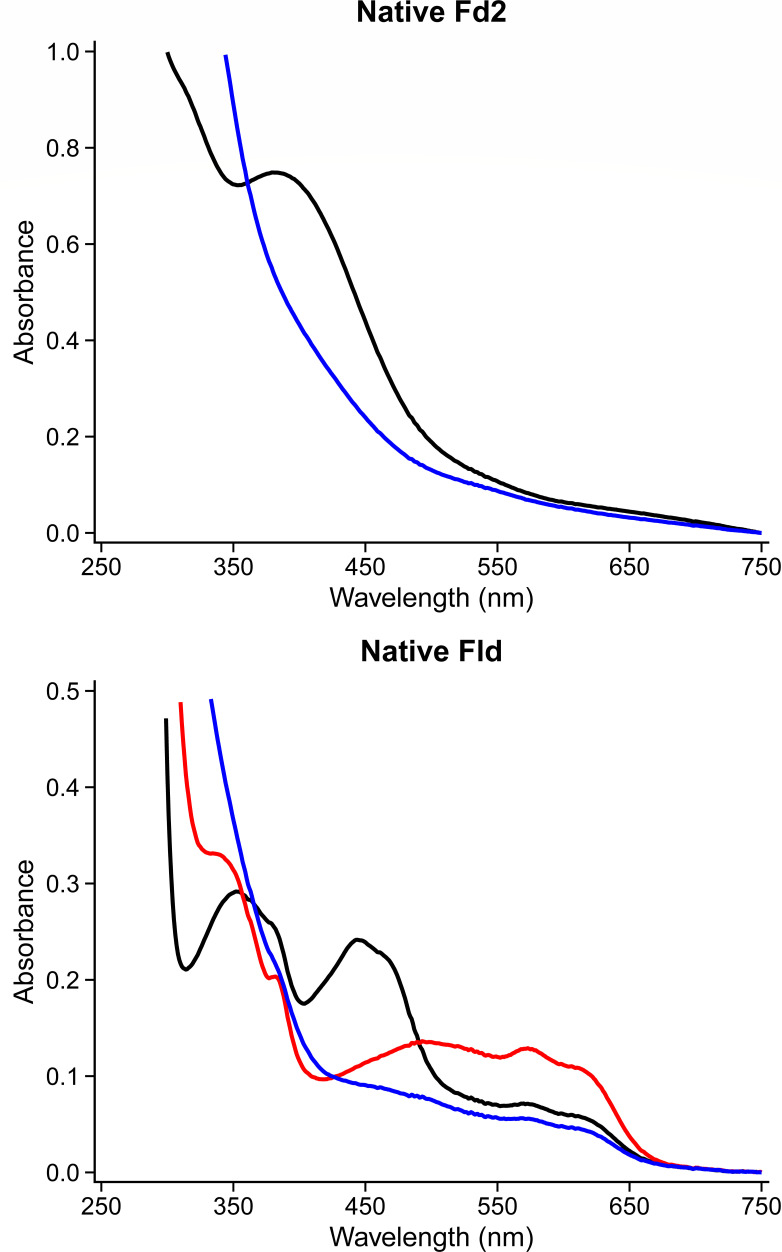
UV-Visible spectra of natively purified Fd2 and Fld. Fd2 and Fld were obtained after HAP and SEC chromatography, respectively, of a cytoplasmic extract of lactate-grown *E. limosum* (Fig. S7). Oxidized (black), semiquinone (red), and reduced (blue) states are shown. Oxidized Fd2 has a characteristic peak at 379 nm, while oxidized Fld has peaks at 352 and 444 nm. A low concentration of the SQ state of Fld is evident by the absorbance between 550 and 650 nm in the oxidized sample.

To purify both the native electron carriers and the oxidoreductases from *E. limosum,* two different approaches were employed (Fig. S6 and S7) in which the cytoplasmic extract was fractionated using either anion exchange chromatography (QHP) or hydroxyapatite chromatography (HAP) as the first step. When a cytoplasmic extract of *E. limosum* cells grown on lactate was fractionated by anion exchange chromatography (QHP), there was no large Fe-containing peak or a peak of absorbance at 390 nm eluting at a high salt concentration characteristic of a single major (acidic) ferredoxin (Fig. S8B). However, as part of the procedure to purify the major oxidoreductases described below, when the extract was fractionated by HAP followed by size exclusion (SEC) chromatography, Fd2 (identified by LC-MS/MS) co-eluted in fractions containing WOR activity, while Fld co-eluted in those containing POR and FDH activities. Although Fd2 co-eluted with WOR from the HAP column, they were separated by SEC, where Fd2 was present in the low molecular weight fractions (Fig. S7C). Fd2 was identified by its size and peak absorbance at 379 nm, and this was confirmed by LC-MS/MS. Fd2 was not detected by SDS-electrophoresis, as is typically the case with Fds. Natively purified Fd2 displayed the same characteristic features in its oxidized and reduced states as heterologously expressed Strep-tagged Fd2 (Fig. 4). Similarly, Fld co-eluted with FDH and POR from a HAP column but was separated as a distinct low molecular weight peak on SEC. It was identified by its absorbance maxima at 352 and 444 nm, and it migrated on an SDS-gel to the expected size (15.2 kDa, Fig. S7B and D). Its identity was confirmed by LC-MS/MS. Purified Fld was identified by its purple color during the purification procedure, indicating that it was partially in a stable semiquinone state (Fig. 4). The natively purified Fld had the same visible absorption properties as the heterologously expressed Strep-tagged protein and exhibited the three characteristic redox states (Fig. 4).

### Partial purification of POR, WOR, and FDH

To investigate the abilities of Fd1, Fd2, and Fld to serve as electron carriers for the native oxidoreductases of *E. limosum*, POR, FDH, and WOR were partially purified from lactate-grown cells using dye-linked activity assays. POR and FDH activities overlapped in the fractions from the HAP column, but WOR was separated (Fig. S7A). The specific activities of the combined fractions of each were comparable at 17.6, 7.1, and 14.4 U/mg for POR, FDH, and WOR, respectively. Similar results were obtained after fractionation on QHP, but in this case, the three enzymes were separated from each other (Fig. S8) with specific activities of 16.5, 14.0, and 11.1 U/mg for POR, FDH, and WOR, respectively. The combined fractions for the enzymes from the HAP (POR + FDH and WOR) and QHP (POR, FDH, and WOR) were further purified by SEC, and while (in the case of the HAP fractions) this led to the identification of Fd2 and Fld, there was a large decrease (>50%) in the recovery of all enzyme activities, suggesting that functional complexes dissociated during the chromatographic fractionation. Separated and partially purified POR, FDH, and WOR from the QHP fractionation were therefore used for investigating electron carrier specificities.

### Oxidoreductase activities with ferredoxins and flavodoxin

We had previously shown that POR is extremely active in *E. limosum* and, moreover, its specific activity increases >10-fold in lactate-grown compared to glucose-grown cells (12). POR catalyzes the low potential oxidation of pyruvate to acetyl-CoA (*E*_m_ = –520 mV). The partially purified POR exhibited a dye-linked activity of 16.5 U/mg and was able to reduce the recombinant forms of Fd1 (25 µM) and Fd2 (25 µM) with specific activities of 6.4 and 3.4 U/mg, respectively. POR also used Fld (25 µM) as an electron acceptor during which the stable intermediate SQ is formed prior to full reduction to HQ. The respective activities were 1.3 (OX to SQ) and 2.2 (SQ to HQ) U/mg (Table 2; Fig. S9). Because POR is a central enzyme in the lactate utilization pathway, we conclude that Fd1, Fd2, and Fld can all be used as electron carriers with comparable activities. Both CODH and FDH in *E. limosum* catalyze the reduction of CO_2_ in the Wood-Ljungdahl pathway either to CO (*E*_m_ = –520 mV) or formate (*E*_m_ = –410 mV), respectively (16). CODH was investigated using the *E. limosum* cytoplasmic extract, which had a dye-linked activity of 18.6 U/mg. CODH oxidized CO and reduced both Fd1 and Fd2 with specific activities of 0.7 and 0.5 U/mg, respectively, while Fld was completely reduced to the HQ state with specific activities of 103 mU/mg (OX to SQ) and 60 mU/mg (SQ to HQ). FDH activity was measured through the reverse reaction where formate is oxidized, and putative electron acceptors are reduced. Partially purified FDH with a dye-linked activity of 7.2 U/mg reduced Fd1 (10 mU/mg) and Fd2 (26 mU/mg), but reduced Fld only to the SQ state (29 mU/mg) but not to the HQ state (<0.1 mU/mg) (Table 2).

**TABLE 2:** POR, FDH, WOR, and CODH activities with Fd1, Fd2, and Fld^*a*^

Enzyme	Dye-linked (U/mg)	Fd1 (mU/mg)	Fd2 (mU/mg)	Fld: Ox to SQ (mU/mg)	Fld: SQ to HQ (mU/mg)
POR	16.5	6,350	3,420	1,290	2,240
FDH	7.2	10	26	29	<0.1
WOR	3.3	35	38	23	2
CODH	18.6	688	469	103	60

^*a*^ Dye-linked activities were obtained using BV as the acceptor. For WOR, the values given are for the non-bifurcating activity.

In *E. limosum*, we previously found that WOR oxidizes various aliphatic and aromatic aldehydes to the corresponding acid in a dye-linked low potential reaction (12). A homologous enzyme has been characterized from the fermentative anaerobe *Acetomicrobium mobile*, whose WOR contains the same five subunits (WorABCSL) as the *E. limosum* enzyme. *A. mobile* WOR exhibiting a dye-linked activity of 64 U/mg was shown to have electron-bifurcating activity where the aldehyde was oxidized to the corresponding acid while *A. mobile* Fd (containing a single [4Fe-4S] cluster like *E. limosum* Fd1) and NAD^+^ were simultaneously reduced with a specific activity of 50 mU/mg based on NAD^+^ reduction (21, 38). The *A. mobile* enzyme did not reduce either NAD^+^ or *A. mobile* Fd if they were the sole electron acceptor. Partially purified *E. limosum* WOR with a dye-linked activity of 3.3 U/mg had extremely low activity with Fd1, Fd2, or Fld as the sole electron carrier (≤30 mU/mg), as expected in the absence of NAD^+^. With Fld, this was with the OX to SQ reaction and no activity (<0.1 mU/mg) was observed for SQ to HQ conversion (Table 2). To test for the bifurcating activity of *E. limosum* WOR, NAD^+^ was added as the last component of the assay in the presence of Fd1, Fd2, or Fld, and the reaction was followed by NADH formation. The results showed that the *E. limosum* enzyme did not reduce Fd and NAD^+^ simultaneously, and only reduced Fd but not NAD. It therefore appears that WOR is no longer functional in terms of its bifurcating activity after partial purification.

### Characterization of *E. limosum* deletion mutants of Fd1, Fd2, and Fld

**Fig 5:**
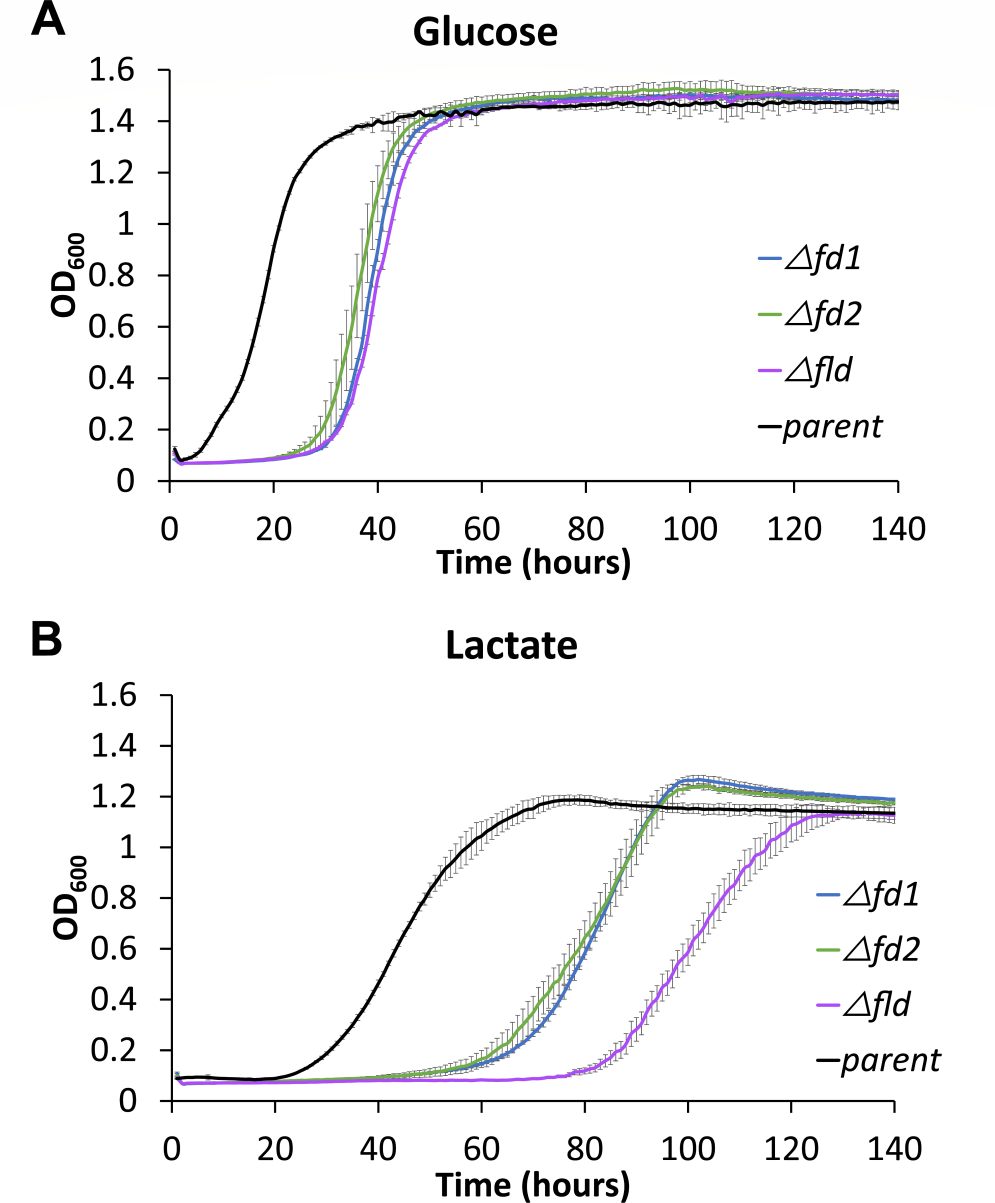
Growth phenotypes of Fd1, Fd2, and Fld deletion mutants compared to the parent strain. Deletion mutants of Fd1, Fd2, and Fld grown on (**A**) glucose and (**B**) lactate in comparison to the parent strain, wild-type *E. limosum,* grown anaerobically at 35°C. The growth curves represent five replicates, and error bars indicate standard deviations.

To further investigate the physiological roles of Fld and the two Fds in *E. limosum*, the genes encoding them were deleted using a linear DNA fragment that had 1 kb homologous regions upstream and downstream of the target gene. This was replaced with an *ermB* marker for selection (Table S2) (44), resulting in *△fd1*, *△fd2*, and *△fld* mutant strains. The parent wild-type strain and the deletion mutant strains were grown on glucose or lactate. On glucose, all three mutants showed a lag phase of about 30 h compared to the parent, although log-phase growth rates and final cell densities were comparable in all strains. These data suggest that glucose metabolism is not dependent on any one of the three redox proteins, and each is likely substituted by the others. However, very different results were obtained on lactate. The 25-h lag phase seen with the parent (a lag is always observed compared to growth on glucose, where the lag is minimal) doubled to about 50 h in the *△fd1* and *△fd2* strains but increased to about 90 h with the *△fld* strain (Fig. 5). In addition, the *△fld* mutant strain appears to grow more slowly than the wild-type parent strain. All mutants were grown in Fe-sufficient, non-limiting conditions (7.7 µM Fe). To determine what Fe-limitation conditions were, wild-type *E. limosum* was grown in glucose and lactate conditions under various added Fe concentrations ranging from 0 to 100 µM. We find that 5 µM Fe added is sufficient for growth, and the 7.7 µM Fe added to the normal media conditions is not a limiting factor. Clearly, Fld plays an important role in lactate but not glucose metabolism, and one that is not substituted by either Fd1 or Fd2.

## DISCUSSION

*E. limosum* has five putative electron acceptors encoded in its genome, two ferredoxins (Fd1 and Fd2) and three flavodoxins (Fld, Fld-like1, and Fld-like2). In terms of the Fds, Fd2 but not Fd1 could be purified from cell extracts. Both Fd1 and Fd2 served as electron carriers for POR but were only partially reduced by FDH. Both Fd1 and Fd2 were reduced by WOR, but in the bifurcating reaction with NAD^+^ as the obligatory second electron carrier, both Fd1 and Fd2 were reduced without the expected strict dependence on NAD^+^ as defined for bifurcating systems (45). As noted above, the bifurcating WOR from *A. mobile* (38) has a measured bifurcating activity that was three orders of magnitude lower than the dye-linked activity. Herein, we show that with *E. limosum,* the dye-linked activity of partially purified WOR was about 20-fold less than that of the *A. mobile* enzyme. Hence, assuming the two enzymes have comparable activities, the expected bifurcating activity of *E. limosum* WOR would have been beyond detection. It seems likely that within the cell, WOR associates with other oxidoreductases and redox proteins to give functional bifurcating multiprotein complexes, and that these are broken up during purification attempts with a corresponding loss of bifurcation activity (38). Indeed, as we show here, during partial purification of the oxidoreductases, Fd2 was associated with fractions containing WOR while Fld was associated with those containing POR and FDH.

In any event, when grown on glucose or lactate, *E. limosum* appears to have no significant preference for one Fd over the other. This is not surprising considering that purified oxidoreductases are not typically specific to the Fd from the same organism, and in various biochemical studies, the readily available Fd from *C. pasteurianum* (equivalent to *E. limosum* Fd2) is often used for *in vitro* assays (17, 18). Accordingly, with *E. limosum,* the *△fd1* and *△fd2* deletion mutants had the same phenotypes, and both exhibited increased lag phases compared to the parent but reached similar final cell densities. In fact, the two mutants had the same phenotype during growth on lactate, with each reaching slightly higher cell densities than the parent after the significant growth lag. These data raise the question of why both Fds are maintained in the cell, considering that from the pangenome analysis, Fd2 is highly conserved, but Fd1 is not (Fig. 2). This might be due to an unknown growth condition that leads to a competitive advantage with the presence of Fd1.

Fld was natively purified from extracts of *E. limosum* cells, and the gene encoding it increased in expression under lactate conditions in comparison to growth on glucose (Fig. 1). The *△fld* mutant also has a clear and strong negative growth phenotype during growth on lactate, suggesting that Fld plays an important role during lactate utilization (Fig. 5). *E. limosum* Fld is a canonical flavodoxin existing in three stable redox states: OX, SQ, and HQ, and clearly provides a low-potential electron carrier equivalent to that of Fd (Fig. 3; Fig. S5). Fld was reduced by two electrons to the HQ state in the POR and CODH reactions (Table 2), consistent with the low redox potentials of pyruvate and CO (both near –510 mV), but FDH only reduced it to the SQ state. In general, the redox potential of the OX/SQ couple in Fld is near −200 mV, while that of the SQ/HQ is much lower and closer to −450 mV, similar to that of Fds (34, 35, 46, 47). Previously characterized FDHs typically oxidize formate (*E*_m_ = –410 mV) and reduce the higher potential electron carrier NAD^+^ (*E*_m_ = −320 mV) (13) or form a bifunctional complex with a bifurcating hydrogenase enzyme (H_2_/H^+^, *E*_m_ = −420 mV) (48). In the case of *E. limosum*, FDH functions in the Wood-Ljungdahl pathway to reduce CO_2_, so it may not be expected to reduce the Fld SQ to the HQ form with formate as an electron donor, as we demonstrated experimentally, or reduce the low-potential Fd1 and Fd2 (Table 2). FDH is most likely reducing NAD^+^, like previously well-characterized enzymes, as opposed to using Fd, as previously predicted for *E. limosum*. In contrast, for the WOR reaction, the RCHO/RCOO^−^ couple has a very low reduction potential near −520 mV (depending on the R group) (49, 50), and would be expected to reduce any of the redox proteins under study. However, WOR lost activity during purification, and its bifurcating activity was too low to be measured. Nevertheless, we show here that two major enzymes in *E. limosum* metabolism, POR and CODH, both utilize all three low-potential electron carriers, Fd1, Fd2, and Fld, in *in vitro* assays.

We previously showed that *E. limosum* has a unique pathway for metabolizing lactate when compared to two other well-studied lactate utilizers in the Clostridia class strains, namely *Acetobacterium woodii* in Group d and *Anaerobutyricum hallii* in Group n (Fig. 2; Fig. S2) (12). Specifically, *A. woodii* only generates acetate (not butyrate) and *A. hallii* produces only butyrate (not acetate) while *E. limosum* converts lactate to both butyrate and acetate (Fig. S10). All three strains were proposed to use LCT and POR to convert lactate to acetyl-CoA. Hence the three Groups to which they belong, d, g, and n, all contain POR and LCT (Fig. 2). POR is central to primary metabolism, and this type of POR is present in 89% of the 350 organisms in the phylogenetic tree, but LCT is more specific to lactate catabolism and is found only in 33% of the tree organisms. However, the LCT/POR pathway is the only strategy common to all three model organisms.

Like *E. limosum*, *A. woodii* uses the Wood-Ljungdahl pathway to convert CO_2_ to acetate, in addition to that produced from lactate via pyruvate, and all members of Groups d and g contain FDH and CODH (Fig. 2), although they are found in only 21% and 28% of all 350 tree organisms, respectively. Like *E. limosum, A. hallii* converts pyruvate-derived acetyl-CoA to butyrate, and all Groups g and n members contain BCD (Fig. 2), which, surprisingly, is found in 63% of all tree organisms, suggesting that butyrate production is common in this *Clostridium* class. Both *A. woodii* and *A. hallii* contain Fd2, which is found in 99% of tree organisms (Fig. 2). Both *A. woodii* and *A. hallii* also contain Fld; in fact, all Group n members and 80% of Group d contain Fld. Given our results with *E. limosum* showing the importance of Fld in lactate metabolism in general, this would suggest that Fld is generally important for organisms that can utilize lactate (Fig. S10). No comment can be made about Fld-like2 as this could not be distinguished from Fld in the pangenome analysis. However, it is interesting that “long” Fld-like1 is also present in *A. hallii* and is found in 87% of Group d and 50% of Group n organisms, but with the exceptions of Groups b, d, and i, it is not very prevalent in the other groups. Clearly, Fld-like1 is indeed a flavodoxin-like redox protein of unknown function in certain types of the 350-member *Clostridium* class depicted in Fig. 2.

The phylogenetic tree of clostridia-like strains (Fig. S2) contained 10 groupings that were primarily composed of the genus *Eubacterium* interspersed between groupings from other genera, classes, and even orders. The *Eubacterium* genus is broadly defined as obligate anaerobic gram-positive non-spore forming rods that lack certain fermentative characteristics (they do not produce propionate, lactate, or succinate as major products during glucose degradation) (51, 52). Defining the *Eubacterium* genus by these negative criteria likely accounts for the wide distribution of the genus, which has involved numerous instances of reclassification of former *Eubacterium* strains to other genera and vice versa (52–55). In fact, based on the phylogenetic tree herein, constructed using 49 universal genes (39), several genomes appear to be misclassified into the wrong genus, including *Roseburia* sp. OM02-15 and *Roseburia* sp. BX1005 within *Eubacterium* Groups t and u, respectively, and *Eubacterium* sp. strain L3_068_000M1, *Eubacterium siraeum* strain MGYG-HGUT-02530, *Eubacterium* sp. C1_bin.40_sub.fa, and *Eubacterium* sp. B73_bin.13.fa within *Ruminococcus* Groups h and i (Fig. S2; Table S1). We found that the distribution of the genes of interest in this study (encoding Fds, Flds, and key oxidoreductases) were highly correlated with branch points in the tree, with 73% of gene/grouping pairs present at either 0% or 100% (Fig. 2). The distribution of defining metabolic genes on trees constructed using universal genes could be a useful tool in the future for disentangling phylogenetic relationships between ill-defined genera and species (39).

Our conclusion from the mutant analysis that Fld rather than Fd (Fd1 or Fd2) is more important in lactate metabolism and SCFA production in *E. limosum* is very surprising given that the organism was grown with more than a sufficient supply of iron (7.7 µM) (Fig. S11). In general, Flds serve an electron transfer role only under conditions of iron limitation when insufficient iron is available to maintain the high levels of Fd required for fermentative metabolism. This has been well documented in species such as *Acidaminococcus fermentans, Peptostreptococcus elsdenii, Clostridium pasteurianum*, and *Anabaena* sp. (33, 34, 56). For *A. fermentans*, under normal iron conditions (7 µM Fe), cells were found to contain a high concentration of Fld and a low concentration of Fd, and this was reversed when a high iron concentration (45 µM Fe) was added (34). Based on our previous metal uptake data with *E. limosum*, which included iron, and the elution of metal-containing proteins from chromatography columns, there is no indication that the organism is limited for iron under standard growth conditions using lactate as the carbon source (12, 38). Indeed, the opposite appears to be the case. We showed that growth on lactate compared to glucose greatly stimulates iron uptake via two iron transporters that are regulated at both the transcriptional and translational levels, including by tungsten-dependent riboswitches (12). The iron content of lactate-grown cells increased more than an order of magnitude compared to those grown on glucose (from 8.8 to 102 µmol/mg of protein). This is consistent with the increased demand for iron for iron-containing oxidoreductases involved in lactate metabolism, notably POR (12 Fe atoms/mole), FDH (18 Fe atoms/mole), CODH (28 Fe atoms/mole), and WOR (46 Fe atoms/mole). Hence, a primary role for Fld in lactate metabolism is even more surprising.

In the human gut environment where *E. limosum* is found, there is fierce competition for iron between the anaerobic microbes as well as with the host (57, 58). Some are well prepared for this battle, such as the pathogen *Clostridium difficile*, which has eight putative Flds in its genome, four of which are “long” Fld-like. Its primary Fld, termed FldX, is a typical Fld, and the gene encoding it is highly expressed. This likely contributes to the organism being a formidable pathogen in the human gut (37). In a similar manner, *E. limosum* may be expressing and utilizing Fld as an electron carrier for lactate metabolism in response to the competitive environment of the human gut, where any means to reduce the requirement for iron provides a competitive advantage. Hence, *E. limosum* is able to use Fd1 and Fd2 as electron carriers for its primary metabolic pathways, but for lactate utilization, it appears to have a strong preference for Fld.

## MATERIALS AND METHODS

### Growth of *Eubacterium limosum*

*Eubacterium limosum* ATCC 8486 was routinely grown anaerobically at 35°C as previously described (12) in 150 mL serum bottles containing 50 mL of medium with shaking at 40 rpm under a headspace of 80% N_2_ and 20% CO_2_. Growth curves were carried out in a Bioscreen C (Thermo Labsystems, Milford, MA, USA) in an anaerobic chamber (Plas Labs, Lansing, MI, USA) with low agitation under an atmosphere of H_2_, CO_2_, and N_2_ (5:5:90). RNA extractions and RT-qPCR were conducted exactly as in Putumbaka et al. (12). Growth studies were routinely carried out using five biological replicates, and error bars indicate standard deviations.

### Comparative genomics

Genomes of the *Eubacteriales* and related orders that encompassed the diverse *Eubacterium* genus were selected based on several criteria to maximize diversity and decrease oversampling leading to sampling bias. Using the Bacterial and Viral Bioinformatics Resource Center (BV-BRC) (59), genomes were filtered selecting those that were from cultured organisms, of good quality and above, and that had CheckM values < 3% contamination and >97% completion (where reported). Oversampling was avoided using species diversity by selecting a maximum of 40 genomes per species and preferentially those with the highest quality metrics. In cases where genomes lacked a species name, genome name structure, and Bio Project Accession were used to avoid replicate genomes. FASTA files of genomes were obtained from the National Center for Biotechnology Information (NCBI) (60) and were uploaded to a KBASE narrative (61) for analysis. The genome sequences were first annotated using the Annotate Multiple Microbial Assemblies with RASTtk (v1.073) app (62) and the quality was rechecked using the Assess Genome Quality with CheckM (v1.0.18) app (63). Genomes that had >3% contamination or <97% completion were discarded. Initially, genomes in the BV-BRC database of the *Eubacterium* genus were evaluated and inserted into a rooted phylogenetic species tree using the Insert Set of Genomes Into SpeciesTree (v2.2.0) app, which uses FastTree 2.0.0 and a set of 49 core genes to infer maximum-likelihood phylogenies (64). The tree was visualized using Interactive Tree of Life v6.8.1 (65). It became apparent that the *Eubacterium* genus consisted of several groups that were separated phylogenetically by organisms from other genera, families, and even orders (Fig. S2). Therefore, the Insert Set of Genomes Into SpeciesTree app was used to identify all of the genera that were phylogenetically interspersed between the groups of the *Eubacterium* genus, and genomes from these genera (maximum of 40 per genus) were added to the analysis using the same criteria as the *Eubacterium* genus genomes. This resulted in a final total of 350 genomes (Table S1). A pangenome of the 350 genomes was constructed using the mOTUpan (v0.3.2) app (66, 67) with MMseqs2. Sequences are aligned and clustered with easy-cluster mode for genes that have >80% coverage and >80% amino acid identity for orthologs. This clustered set of genes is defined as a feature which was then utilized to identify proteins.

### Heterologous expression and purification of recombinant Fds and Flds

The genes encoding Fd1 (B2M23_RS10700) and Fd2 (B2M23_RS02975) were cloned into pET-21a(−) plasmid (Novagen) under the control of T7 promoter. That of Fd1 was unmodified, while that encoding Fd2 also encoded a C-terminal Strep II tag. The plasmids were transformed into *E. coli* Rosetta BL21(DE3) *ΔiscR*, and single-colony transformants were grown aerobically in 2 L LB medium supplemented with 100 μg/mL ampicillin at 37°C with shaking at 220 rpm until an OD_600_ of 0.6 was reached. Ferric ammonium citrate (2 mM) and l-cysteine (2 mM) were added, and the cultures were cooled down to 25°C. Glucose (0.5%, wt/vol) and sodium fumarate (10 mM) were added, and the cultures were maintained anaerobically. Induction of protein expression was initiated by adding 0.5 mM isopropyl β-d-1-thiogalactopyranoside (IPTG), and the bottles were tightly sealed, followed by overnight incubation at 25°C with shaking at 180 rpm. Cells were harvested by centrifugation at 7,000 × *g* for 10 min at 4°C and subsequently transferred to a Coy anaerobic chamber containing 5% H_2_ (95% Ar). Genes encoding Fld (B2M23_RS20265), Fld-like1 (B2M23_RS01705), and Fld-like2 (B2M23_RS10125) were individually cloned into pET-24a (+) plasmid (Novagen), each with a C-terminal Strep II tag under the control of T7 promoter. Each expression vector was transformed into *E. coli* Rosetta BL21(DE3) (Novagen), and single-colony transformants were cultured in 2 L LB medium with 50 μg/mL kanamycin at 37°C with shaking at 220 rpm until an OD_600_ of 0.6 was reached. Riboflavin, FMN (2 mM), FAD (2 mM), glucose (0.5% wt/vol), and sodium fumarate (10 mM) were added, and gene expression was induced by adding 0.5 mM IPTG, followed by overnight incubation at 25°C with shaking at 180 rpm. Cells were harvested by centrifugation at 7,000 × *g* for 10 min at 4°C and transferred to the Coy anaerobic chamber. All subsequent purification steps were performed anaerobically.

Cells were resuspended in 50 mM HEPES, pH 7.5, containing 1 mM MgCl_2_, 2 µM FMN, 2 µM FAD, 1 mM cysteine, and lysed by sonication in the presence of lysozyme (0.5 mg/mL) and DNase I (0.1 mg/mL). A cytoplasmic extract was prepared by centrifugation at 35,000 × *g* for 1 h at 10°C. For the purification of recombinant Fd1, anion exchange chromatography was performed using a 5-mL Q Sepharose Fast Flow (QFF) column equilibrated with 50 mM HEPES, pH 7.5. Bound proteins were eluted with a linear gradient from 0 to 500 mM NaCl in the same buffer. Size exclusion chromatography was performed using a HiLoad Superdex 200 prepgrade XK 16/60 column (Cytiva) with a running buffer of 50 mM HEPES, pH 7.5, at a flow rate of 1.25 mL/min. For the purification of recombinant Fd2, Fld, Fld-like1, and Fld-like2, the supernatant was mixed with 5 mL of Strep-Tactin Superflow Plus resin (IBA Lifesciences, Göttingen, Germany), equilibrated with 50 mM HEPES, pH 7.5, 1 mM MgCl_22_, 2 µM FMN, 2 µM FAD, and 1 mM cysteine. After incubation at 4°C for 1 h with shaking, it was applied to a 20 mL gravity flow column. Proteins were eluted with the same buffer supplemented with 2.5 mM desthiobiotin. Fractions containing Fd2 and the flavodoxins were concentrated using an Amicon Ultra centrifugal filter (Millipore, 10 kDa molecular weight cutoff) in the same buffer. UV-visible absorption spectra were recorded in an Agilent Cary 3500 UV/Vis spectrometer while enzymes were reduced with Ti(III)citrate. Reconstitution attempts of Fld-like1 and Fld-like2 were carried out by incubating the protein for at least 2 h in 25 mM HEPES buffer, pH 7.5, containing 1 mM MgCl_2_ and 1 mM FMN for Fld-like1 and 1 mM FMN plus 1 mM FAD for Fld-like2. Each protein was then loaded onto a 5 mL QHP column (Cytiva) and eluted with 500 mM NaCl in the same buffer to remove the unbound FMN/FAD and to concentrate the proteins.

### Purification of oxidoreductases from *E. limosum*

In an anaerobic chamber (5% H_2_, 95% Ar), 20 g of frozen lactate grown *E. limosum* cells were thawed and resuspended in 40 mL of lysis buffer containing 50 mM HEPES, pH 7.5, 5% trehalose (wt/vol), 1 mM cysteine, 1 µM FMN, 1 µM FAD, and 100 mg/L deoxyribonuclease (DNase) I. Cells were lysed on ice by sonication (30-s intervals, amplitude 80; model Q55; Qsonica, Newtown, CT, USA). A cytoplasmic extract was prepared by ultracentrifugation at 100,000 × *g* for 1 h at 10°C. Anion exchange chromatography was carried out using a 5 mL Q Sepharose high-performance (QHP) column pre-equilibrated with 25 mM HEPES, pH 7.5 containing 1% (wt/vol) trehalose, 1 mM cysteine, 1 µM FMN, and 1 µM FAD. Bound proteins were eluted with a linear gradient from 0 to 500 mM NaCl in the same buffer at a flow rate of 5 mL/min. Fractions were collected in Ar-flushed serum vials sealed with rubber stoppers and were stored at 4°C. Cytoplasmic extract was also run down a custom 70 mL hydroxyapatite (HAP) column (Bio-Rad) calibrated with 25 mM HEPES, pH 7.5 containing 1% (wt/vol) trehalose, 1 mM cysteine, 1 µM FMN, and 1 µM FAD. Fractions were eluted with a 0–500 mM sodium phosphate gradient, pH 7.5. Fractions were collected in Ar-flushed serum vials sealed with rubber stoppers and were stored at 4°C. In both cases, all fractions were assayed for POR, FDH, and WOR activities. Multiple fractions with the same protein activity were pooled together, concentrated, and applied to a HiLoad Superdex 200 prepgrade XK 16/60 column (Cytiva) with a running buffer of 25 mM HEPES, pH 7.5, containing 250 nM NaCl and 1% trehalose at a flow rate of 1.25 mL/min. The Bradford protein estimation reagent (Bio-Rad) was used to quantify the amounts of protein at all steps.

### Oxidoreductase activity assays

To conduct enzymatic assays anaerobically, all buffers were degassed through cycles of vacuum and sparging with argon gas, and stored in an anaerobic chamber. Once the buffer was added to cuvettes and immediately prior to conducting the reactions, the cuvettes were sealed and then flushed with argon gas. All other reagents for the reaction were added using glass syringes. Dye-linked assays were measured by following the reduction of benzyl viologen (BV) at 600 nm (*ε* = 7,400 cm^−1^⋅M^−1^). Prior to conducting the reaction, the cuvettes were flushed with argon and all reaction components were added using gas-tight syringes. The reaction mixture contained 2 mL buffer, 1 mM BV at 35°C. The reaction was initiated for WOR by the addition of benzaldehyde (250 µM), for FDH by the addition of 5 mM formate, and POR by the addition of a mixture of 5 mM pyruvate, 250 µM CoA, and 250 µM TPP. The stock solutions for benzaldehyde (50 mM) were made in 95% ethanol. One unit of activity using BV as the electron acceptor is defined as the reduction of 1 µmole BV/min. For activity assays with Fd1 and Fd2, their reduction was measured at 425 nm (*ε* = 13,000 cm^−1^⋅M^−1^), while for Fld and Fld-like1, reduction of FMN was measured at 450 nm (*ε* = 10,200 cm^−1^⋅M^−1^), and SQ production and reduction were measured at 620 nm (*ε* = 4,850 cm^−1^⋅M^−1^) (28, 46). In the case of the electron bifurcation activity of WOR, this was followed by the NAD^+^-dependent reduction of Fd at 425 nm (*ε* = 13,000 cm^−1^⋅M^−1^). One unit of activity using the redox proteins as the acceptor is defined as the reduction of 1 µmole of redox protein/min.

### Construction of the *△fd1*, *△fd2,* and *△fld* deletion mutants

To delete the genes encoding Fd1 (B2M23_RS10700), Fd2 (B2M23_RS02975), and Fld (B2M23_RS20265), linear DNA fragments (3 kb) were constructed using NEBuilder HiFi assembly master mix. Each fragment included the *ermB* resistance gene (~1 kb), flanked by ~1 kb homologous sequences corresponding to the upstream and downstream regions of the respective Fd or Fld genes (44) using the primers listed in Table S2. Linear DNA was cleaned using the Zymo DNA Clean & Concentrator kit. The constructed DNA fragments were transformed into the wild-type ATCC 8486 at a concentration greater than or equal to 1 μg of DNA within 5 μL. Competent *E. limosum* cells were made and electroporated following previously published protocols (68, 69). The transformants were recovered for 12 h at 35°C and then plated onto RCM plates containing 1 μg/mL clarithromycin. Colonies containing the deletion strain were confirmed by colony PCR and sequencing.

## SUPPLEMENTAL MATERIAL

Supplemental material aem.00643-26-s0001.pdf Table S2; Fig. S1 to S11.

Table S1 aem.00643-26-s0002.xlsx Selected strains used for comparative genomics.

ASM does not own the copyrights to Supplemental Material that may be linked to, or accessed through, an article. The authors have granted ASM a non-exclusive, world-wide license to publish the Supplemental Material files. Please contact the corresponding author directly for reuse.
