# Supplementary material for: The human gut microbe Eubacterium limosum utilizes flavodoxin over ferredoxin for lactate metabolism
Source: Appl Environ Microbiol. 2026 Jun 26;92(7):e00643-26. doi: 10.1128/aem.00643-26 (PMC13390469; doi:10.1128/aem.00643-26)
Supplement: Supplemental material — Table S2; Fig. S1 to S11. [file aem.00643-26-s0001.pdf]

Supplementary Information

The Human Gut Microbe *Eubacterium limosum* Utilizes Flavodoxin Over Ferredoxin for Lactate Metabolism

Saisuki Putumbaka, Nana Shao, Aaron Donaghy, Emma G. Harrison, Farris L. Poole II, Michael P. Thorgersen, Gerrit J. Schut and Michael W. W. Adams.

Supplementary Tables S1-S2

Supplementary Figures S1-S11

†Address correspondence to adamsm@uga.edu

Corresponding Author:
Michael W. W. Adams
(ORCID ID: 0000-0003-0881-8870)
Department of Biochemistry & Molecular Biology
University of Georgia
Athens, GA 30602, USA
Email: adamsm@uga.edu
Phone: 706 540 1645

Table S1. Strains selected for comparative genomic analysis. A total of 350 different strains were used. Details on how the strains were isolated and genome name assembly information and completeness are provided, together with taxonomic information for each strain and how they were divided into Groups a-x (see **Figure S2**). These data are supplied as a separate excel file.

Table S2. Primers used for constructing deletion mutant strains. All primers used in the construction of deletion mutants and their target regions are listed.

Gene	Primer number	Primer Sequence	Note
fd1	NS107	TGTTTTTTTATTTATATGTTCCTGG	Upstream homologous arm (forward)
	NS108	AAACCACAAAGATATTCAATCCTTTCTGAATC	Upstream homologous arm (reverse) with ermB overlap
	NS111	CTTGCAAACAGCAGGAAACGACCGGTAG	Downstream homologous arm (forward) with ermB overlap
	NS112	ATCCCGGCACCTGGTATACT	Downstream homologous arm (reverse)
fd2	NS113	CGATTTCATCAAGGAACAGC	Upstream homologous arm (forward)
	NS114	AAACCACAAATAAGAATACAAAAAGAAAACACC	Upstream homologous arm (reverse) with ermB overlap
	NS117	CTTGCAAACATTAATACACCCCTTTCTGAAC	Downstream homologous arm (forward) with ermB overlap
	NS118	CTATTAAAAATATAAATTACAGGAGATACTATAGATGGC	Downstream homologous arm (reverse)
fld	NS131	ATGCAGCCAATCAGATGAAGATTATCG	Upstream homologous arm (forward)
	NS132	AAACCACAAATTTTACAGGCCGGCAGAG	Upstream homologous arm (reverse) with ermB overlap
	NS135	CTTGCAAACATTTTATTCTCCTTTAATGATTTTATCAAC	Downstream homologous arm (forward) with ermB overlap
	NS136	AGTGACATTATCACCAATAACAAAT	Downstream homologous arm (reverse)
fld-like1	NS119	GACGCCATGAAAGCCGCC	Upstream homologous arm (forward)
	NS120	AAACCACAAATTAAAAAATCCTCCTTGTATGTTTTGATTGACTTGTTTC	Upstream homologous arm (reverse) with ermB overlap
	NS123	CTTGCAAACATGCCGTTGAGAGTAGCTG	Downstream homologous arm (forward) with ermB overlap
	NS124	GGCACTGGCTATTCCACTCT	Downstream homologous arm (reverse)
Fld-like2	NS113	CGATTTCATCAAGGAACAGC	Upstream homologous arm (forward)
	NS114	AAACCACAAATAAGAATACAAAAAGAAAACACC	Upstream homologous arm (reverse) with ermB overlap
	NS117	CTTGCAAACATTAATACACCCCTTTCTGAAC	Downstream homologous arm (forward) with ermB overlap
	NS118	CTATTAAAAATATAAATTACAGGAGATACTATAGATGGC	Downstream homologous arm (reverse)

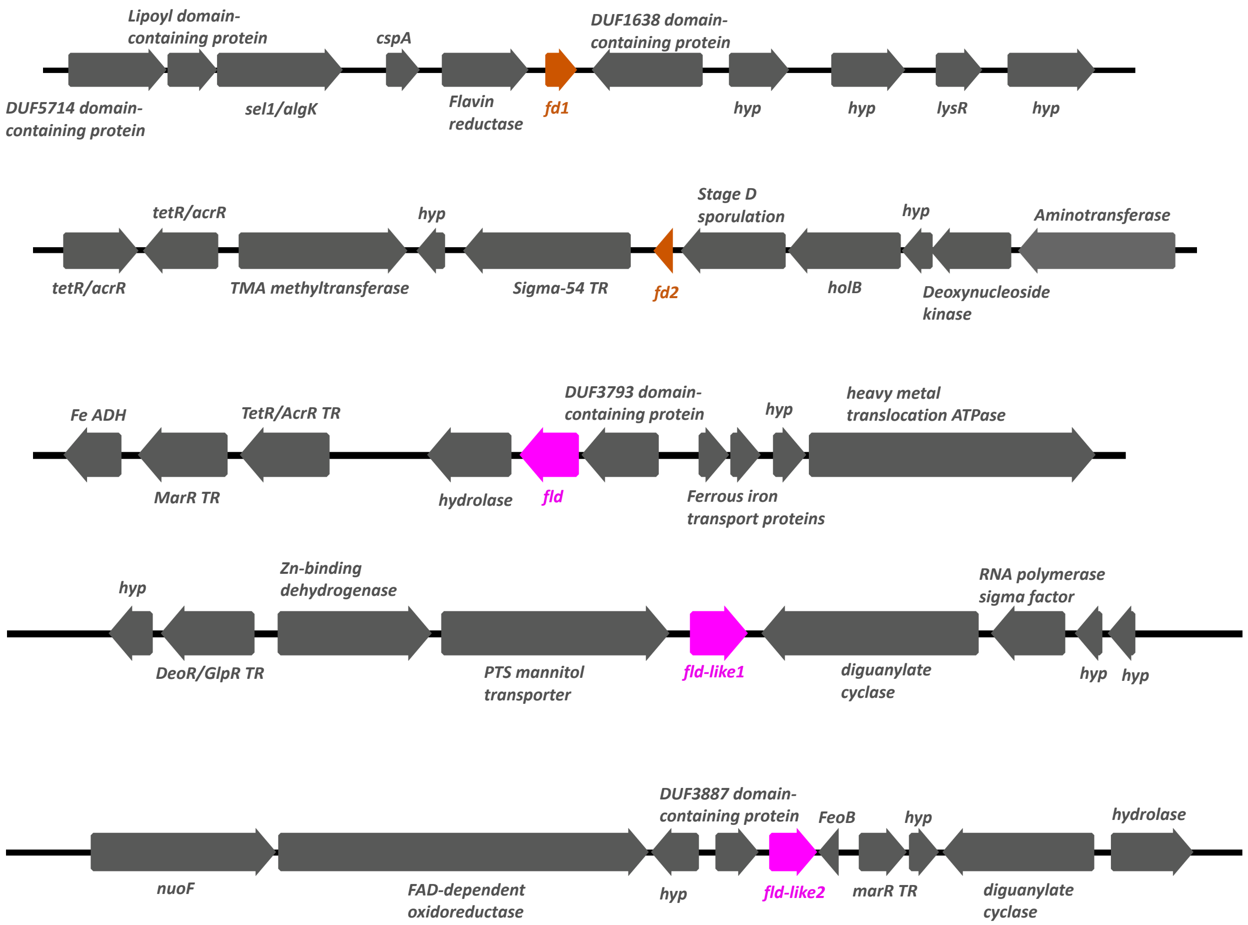

Figure S1. Genome context of Fd1, Fd2, Fld, Fld-like1 and Fld-like2 in the *E. limosum* genome. Fd1 (B2M23_RS10700), Fd2 (B2M23_RS02975), Fld (B2M23_RS20265), Fld-like 1 (B2M23_RS01705), and Fld-like 2 (B2M23_RS10125) gene context with distances from nearby genes are shown relative to each other. Neighboring genes are labeled based on NCBI and Interpro analysis.

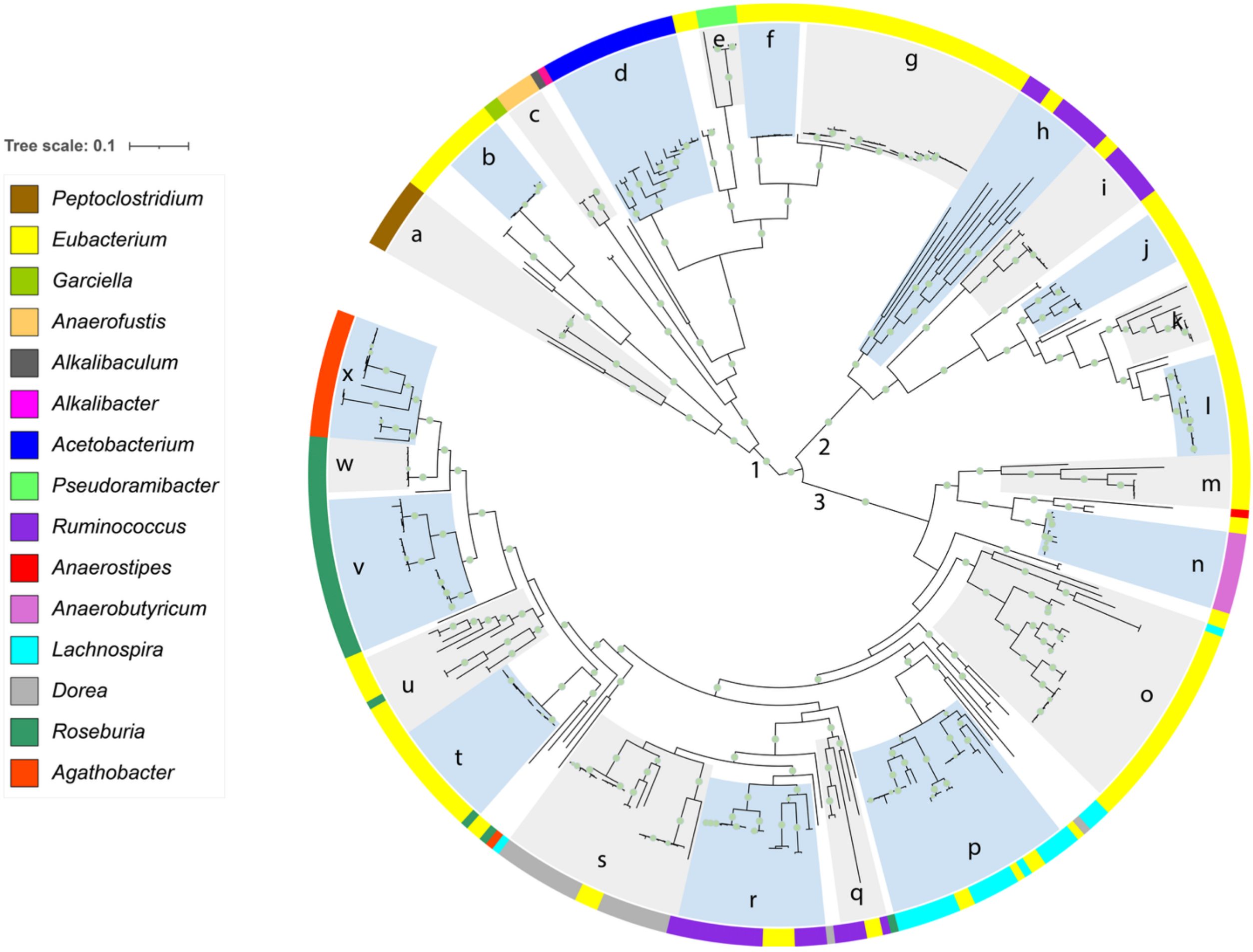

Figure S2. **Phylogenetic tree of clostridia-like strains related to *E. limosum*.** Phylogenetic tree of 350 clostridia strains that encompass the *Eubacterium* genus from the orders Eubacteriales, Peptostreptococcales and Lachnospirales was constructed using multiple sequence alignments of COGs for 49 core universal genes. The ring is colored to denote different genus identifications for the strains as denoted in the key. The tree splits into three main branches as designated by the numbers 1-3. Selected species are highlighted in blue and gray to designate separate Groups (a – x) used in **Table 2**.

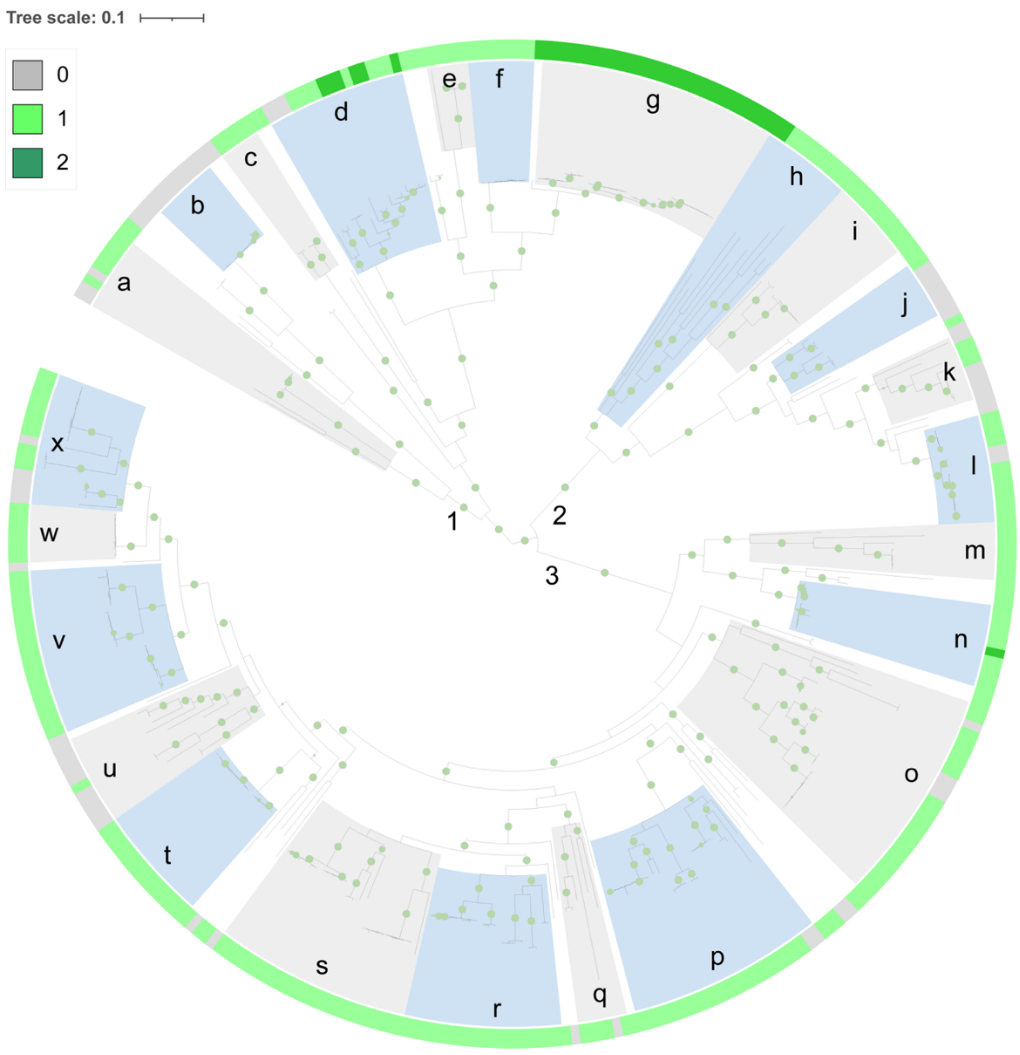

Figure S3: Distribution of *fld/fld-like2* genes in clostridia-like strains. Phylogenetic tree of 350 clostridia strains that encompass the *Eubacterium* genus from the orders Eubacteriales, Peptostreptococcales and Lachnospirales was constructed using multiple sequence alignments of COGs for 49 core universal genes. The ring is colored to denote the number of genes each organism has that fit within the *fld/fld-like2* pangenome feature (grey for zero, light green for one gene, and dark green for two genes). Selected branches are highlighted in blue and gray for Groups a – x for use in **Table 2**.

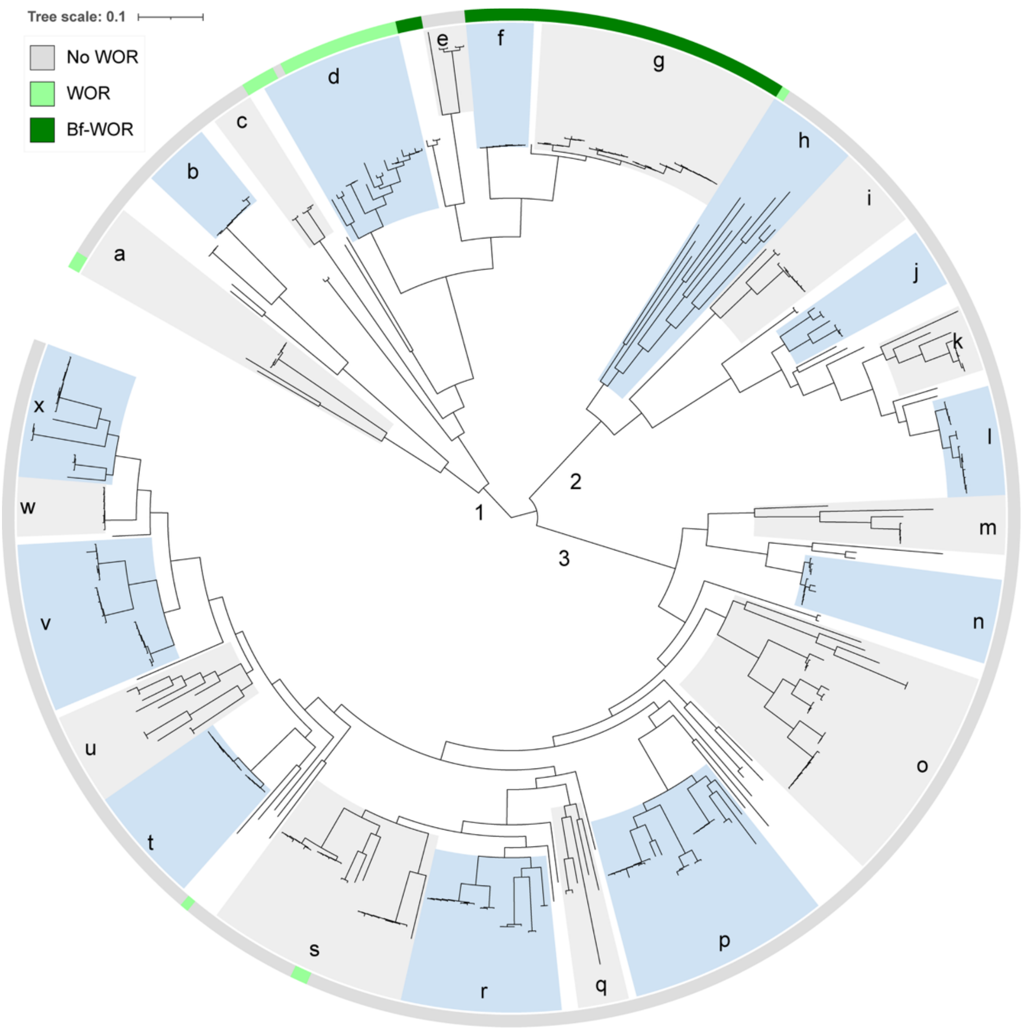

Figure S4: Distribution of *wor* genes in bifurcating (Bf-WOR) and non-bifurcating (WOR) WORs in clostridia-like strains. Phylogenetic tree of 350 clostridia strains that encompass the *Eubacterium* genus from the orders Eubacteriales, Peptostreptococcales and Lachnospirales was constructed using multiple sequence alignments of COGs for 49 core universal genes. The ring is colored to denote the organisms that have genes encoding for non-bifurcating WORs (light green), bifurcating WORs (dark green) and no WORs (grey). Selected branches are highlighted in blue and gray for Groups a – x for use in **Table 2**.

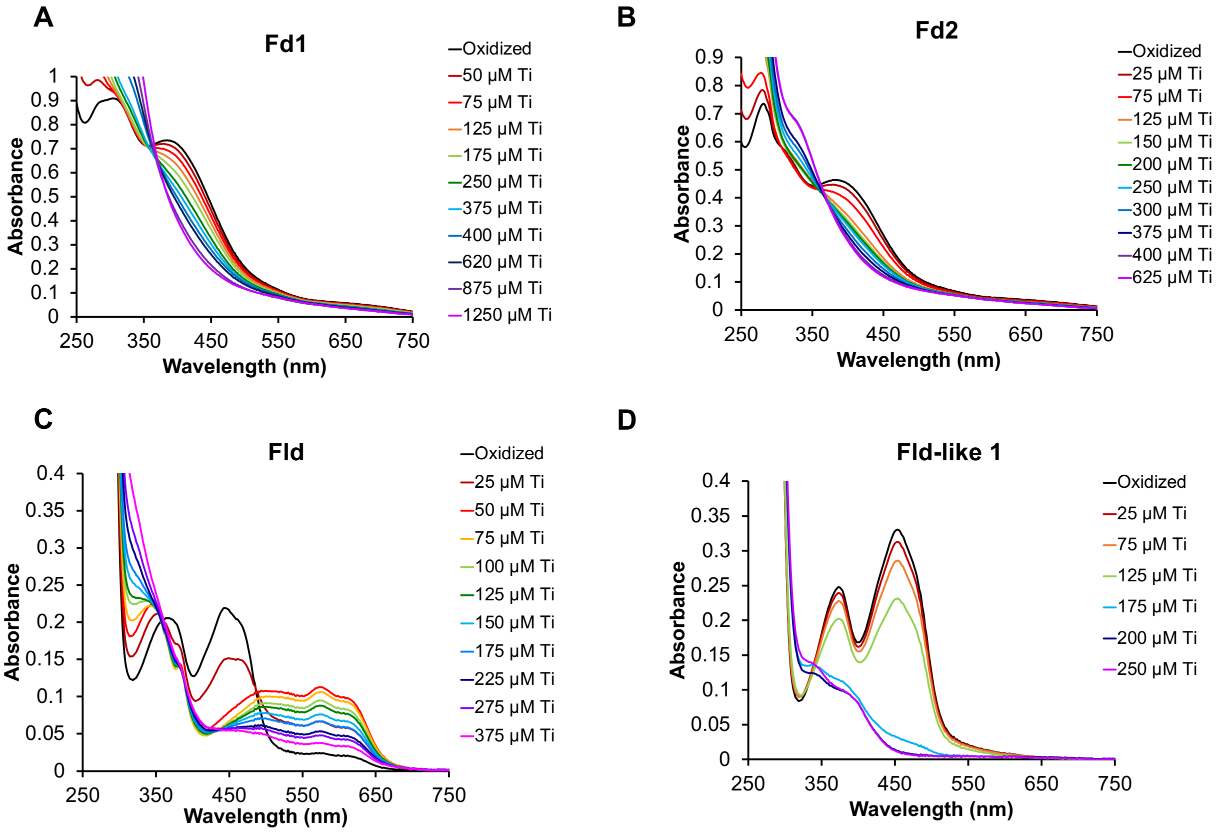

Figure S5. UV-Vis spectra of purified recombinant Fd1, Fd2, Fld and Fld-like1. The oxidized forms of the proteins (25 μM; black lines) were used as purified directly from *E. coli* and these were titrated with the indicated concentrations of Ti (III) citrate.

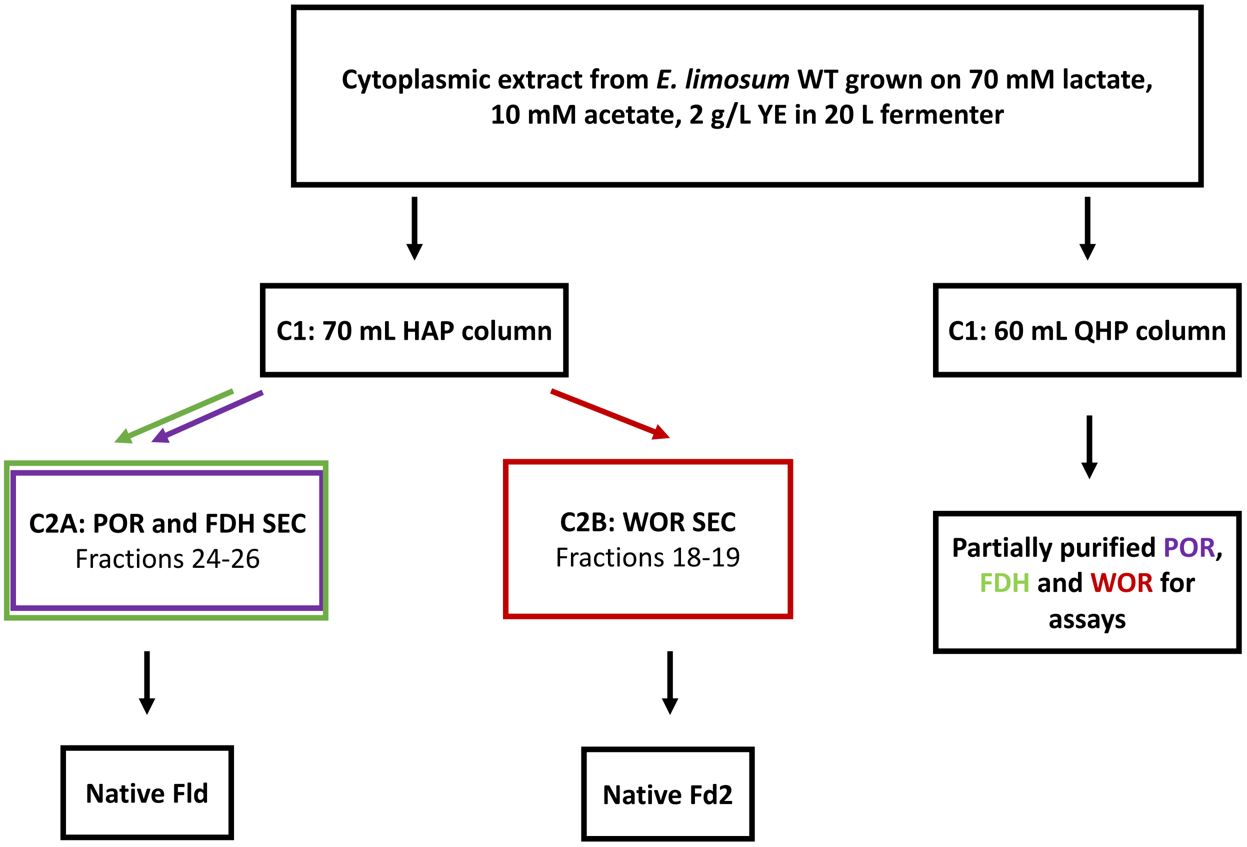

Figure S6. Purification scheme for POR, FDH, WOR, Fd2 and Fld. The cytoplasmic extract from *E. limosum* was fractionated either by HAP or QHP chromatography. From the HAP column, we purified native Fld and Fd2, as depicted in **Figure S7,** and from the QHP column, we obtained partially purified oxidoreductases that were used for enzyme assays as shown in **Figure S8**.

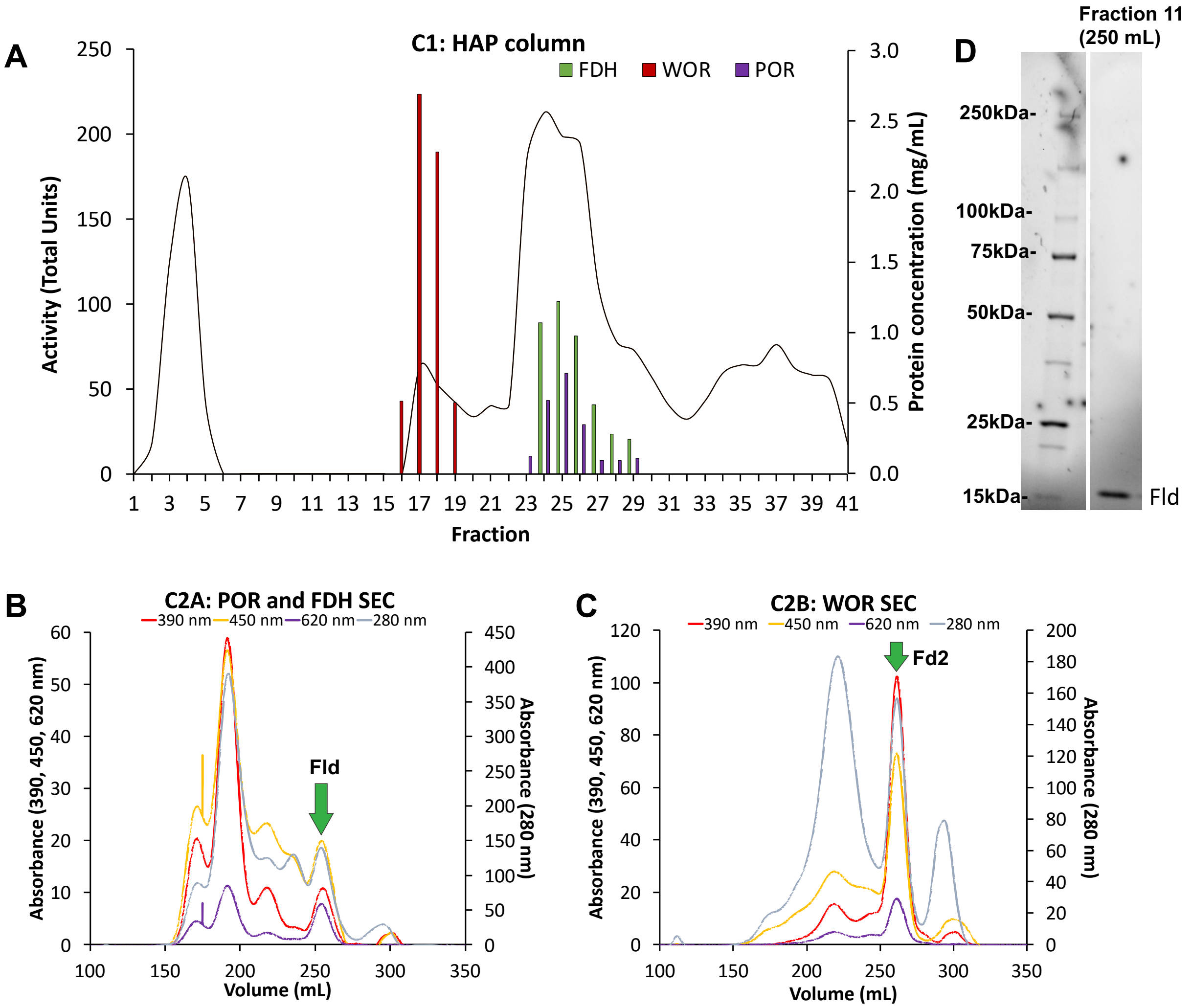

Figure S7. HAP Purification scheme. A) WOR (red line), FDH (green) and POR (purple) dye-linked activities of fractions and protein concentrations in each fraction (black) from the HAP column. B) FDH and POR size exclusion chromatography (SEC) column and C) WOR SEC column. For both SEC runs, the wavelengths measured were for Fe-S clusters at 390 nm (red), for FMN at 450 nm (yellow), for semiquinone at 620 nm (purple) and for protein at 280 nm (grey). D) The gel image of fraction 11 eluting at 250 mL from the SEC column showing native Fld at ~15 kDa. Purified Fd2 was not visible on a gel at the expected size (6 kDa).

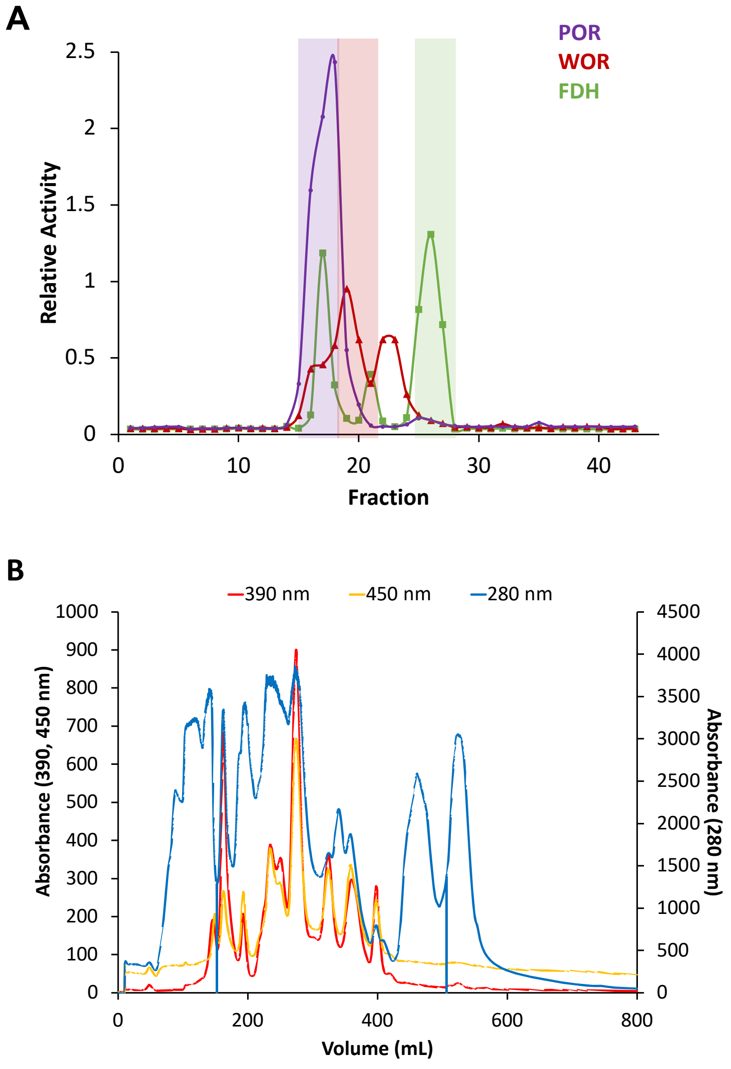

Figure S8. Elution profiles after fractionation of a cytoplasmic extract by anion exchange chromatography (QHP). A) Relative activities of each fraction measured using a dye-linked plate assay. The fractions that were combined for POR (purple line), WOR (red) and FDH (green) and utilized for activity assays with the Fds and Fld are highlighted. B) Absorbance of eluted fractions for iron-sulfur clusters at 390 nm (red), for FMN at 450 nm (yellow) and for protein at 280 nm (blue).

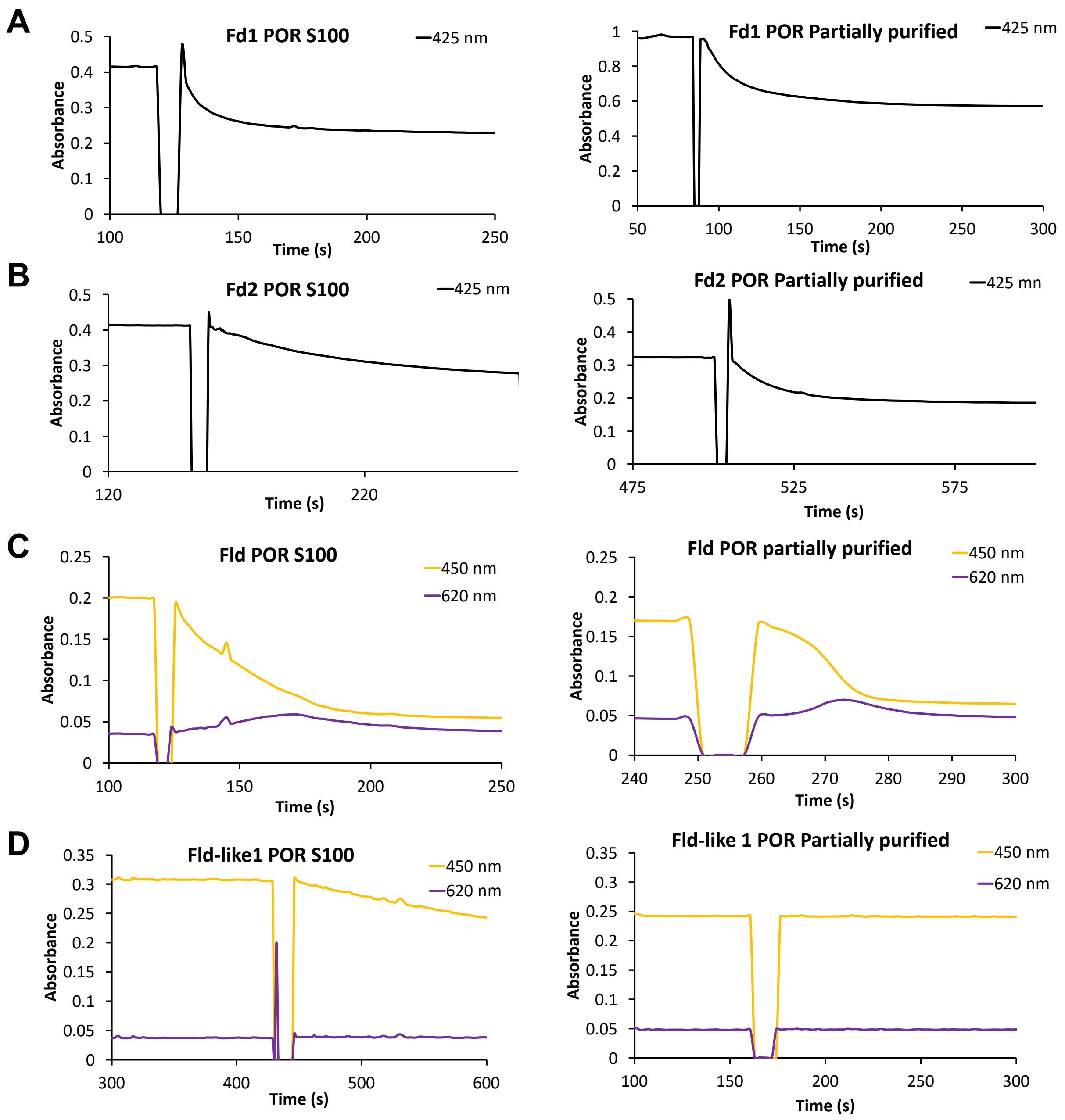

Figure S9. Reduction of A) Fd1, B) Fd2, C) Fld, and D) Fld-like1 by POR using a cytoplasmic extract (S100, *left*) or the partially purified enzyme (*right*). For Fd1 and Fd2, the reduction of [4Fe-4S] clusters is measured at 425 nm. For Fld and Fld-like1, FMN reduction was measured at 450 nm and semiquinone formation and subsequent reduction to the hydroquinone was measured at 620 nm.

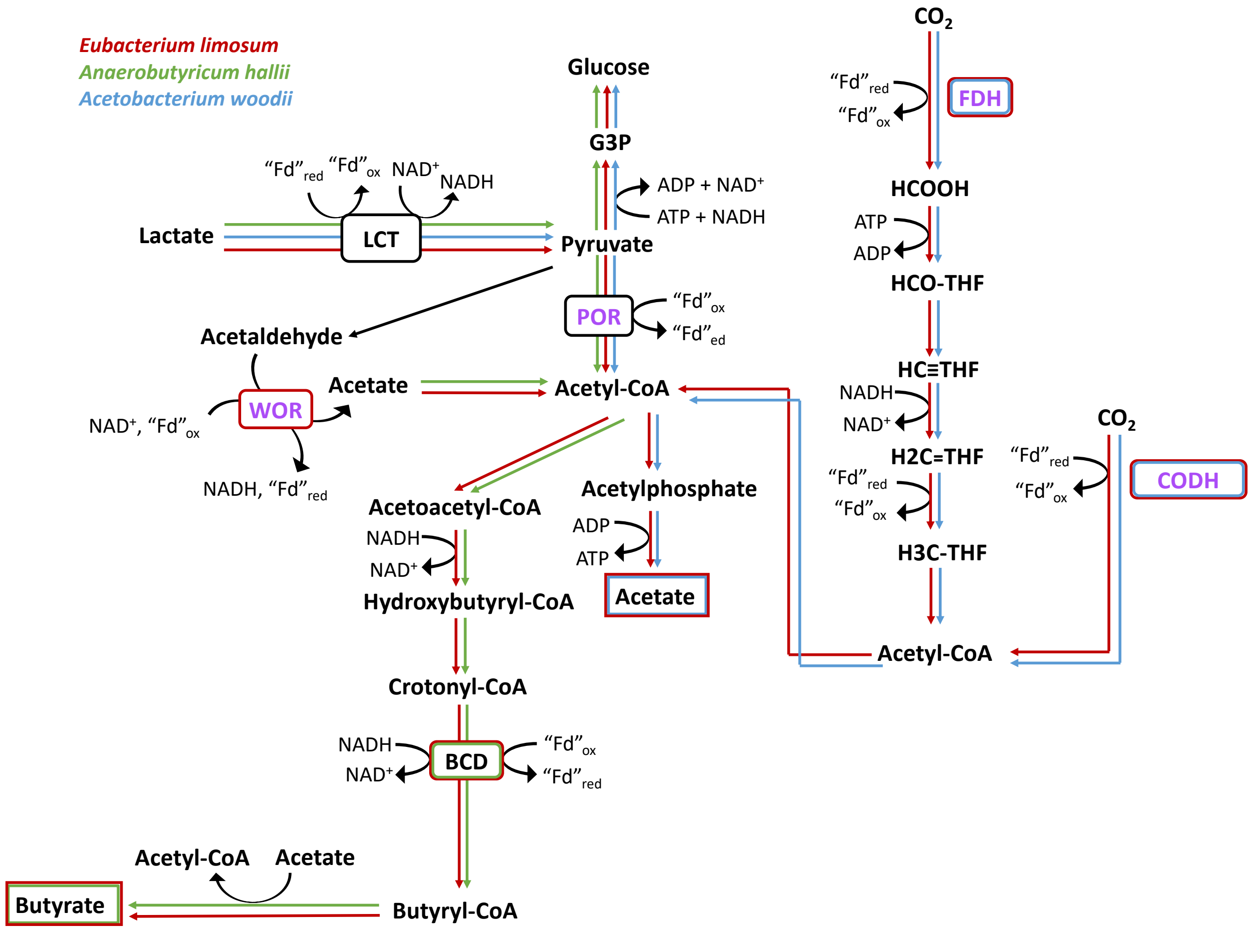

Figure S10. Pathways for lactate utilization in three anaerobic bacteria. Comparison of the pathways used to convert lactate to acetate in *A. woodii*, lactate to butyrate in *A. hallii* and lactate to both butyrate and acetate in *E. limosum.* All enzymes in this pathway can use Fd1, Fd2 or Fld (as designated by "Fd"). The enzymes that were experimentally assayed in *E. limosum* are in purple. Modified from (12).

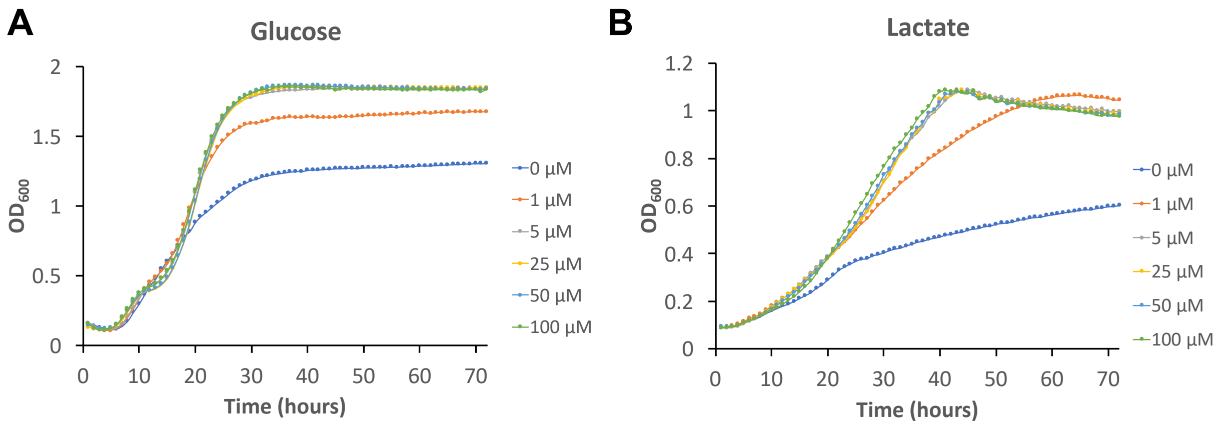

Figure S11. ***E. limosum*** **wild type growth on varying Fe concentrations under glucose and lactate conditions.** The Fe concentrations listed are concentrations of ferrous ammonium citrate added to base *E. limosum* media made with no Fe added. Adding 5 µM Fe to both glucose and lactate growth appears to be enough for sufficient growth and for non-limiting conditions.
